# Tuning nanofiltration membrane performance: OH–MoS_2_ nanosheet engineering and divalent cation influence on fouling and organic removal

**DOI:** 10.1186/s11671-023-03909-2

**Published:** 2023-10-23

**Authors:** Deepak Surendhra Mallya, Guoliang Yang, Weiwei Lei, Shobha Muthukumaran, Kanagaratnam Baskaran

**Affiliations:** 1 School of Engineering, Deakin University, Waurn Ponds, Geelong, VIC 3216 Australia; 2 Institute of Frontier Materials, Deakin University, Waurn Ponds, Geelong, VIC 3220 Australia; 3 Institute for Sustainability Industries and Liveable Cities, Victoria University, Melbourne, VIC 3011 Australia; 4 College of Sport, Health and Engineering, Victoria University, Melbourne, VIC 3011 Australia

**Keywords:** Natural organic matter, Nanofiltration membranes, Organic fouling, MoS_2_ nanosheet, Calcium ion, Magnesium ion

## Abstract

Natural organic matter (NOM) present in surface water causes severe organic fouling of nanofiltration (NF) membranes employed for the production of potable water. Calcium (Ca^2+^) and magnesium (Mg^2+^) are alkaline earth metals present in natural surface water and severely exacerbate organic fouling owing to their ability to cause charge neutralization, complexation, and bridging of NOM and the membrane surface. Hence, it is of practical significance to engineer membranes with properties suitable for addressing organic fouling in the presence of these cations. This study employed OH-functionalized molybdenum disulphide (OH–MoS_2_) nanosheets as nanofillers via the interfacial polymerization reaction to engineer NF membranes for enhanced removal of NOM and fouling mitigation performance. At an optimized concentration of 0.010 wt.% of OH–MoS_2_ nanosheet, the membrane was endowed with higher hydrophilicity, negative charge and rougher membrane morphology which enhanced the pure water permeance by 46.33% from 11.2 to 16.39 L m^−2^ h^−1^ bar^−1^ while bridging the trade-off between permeance and salt selectivity. The fouling performance was evaluated using humic acid (HA) and sodium alginate (SA), which represent the hydrophobic and hydrophilic components of NOM in the presence of 0, 0.5, and 1 mM Ca^2+^ and Mg^2+^, respectively, and the performance was benchmarked with control and commercial membranes. The modified membrane exhibited normalized fluxes of 95.09% and 93.26% for HA and SA, respectively, at the end of the 6 h filtration experiments, compared to the control membrane at 89.71% and 74.25%, respectively. This study also revealed that Ca^2+^ has a more detrimental effect than Mg^2+^ on organic fouling and NOM removal. The engineered membrane outperformed the commercial and the pristine membranes during fouling tests in the presence of 1 mM Ca^2+^ and Mg^2+^ in the feed solution. In summary, this study has shown that incorporating OH–MoS_2_ nanosheets into membranes is a promising strategy for producing potable water from alternative water sources with high salt and NOM contents.

**Supplementary Information:**

The online version contains supplementary material available at 10.1186/s11671-023-03909-2.

## Introduction

With the increased demand for potable water, new membrane-based water purification materials are being extensively developed [1]. Nanofiltration (NF) membranes are promising single-step treatment methods that offer a resourceful approach to meet multiple water quality guidelines and facilitate the removal of natural organic matter (NOM), inorganic matter, micropollutants, heavy metals, and microorganisms during potable water production [1, 2]. In general, NF membrane separation performance is primarily governed by a combination of membrane properties, including electrostatic interactions, size exclusion, and hydrophobic/hydrophilic interactions, as well as the feed water chemistry and filtration conditions such as pressure, crossflow velocity, and temperature [3]. NF membranes have been widely used for potable water production from groundwater and surface water sources [4]. Although NF membranes have demonstrated superior performance compared to conventional treatment alternatives, such as coagulation, adsorption, and medium filtration, membrane fouling remains a significant challenge for water treatment facilities [5]. Significant research efforts have focused on understanding the fouling mechanisms, membrane–foulant interactions, and influencing factors, as well as the synthesis of next-generation engineered membranes for fouling mitigation [6, 7].

Organic fouling is primarily caused by the abundance of natural organic matter (NOM) in the water sources. NOM is problematic because it causes pore blockage of the membranes, significantly reducing permeance and deteriorating separation performance, as well as membrane structural integrity [6]. Furthermore, during the disinfection stage of water treatment, NOM reacts with disinfectants to form a suite of carcinogenic disinfection by-products (DBPs) [8]. NF membranes have proven to be very effective in removing various NOM fractions and supporting the production of safe drinking water [5]. A commercial XN45 NF membrane removed > 70% of NOM as dissolved organic carbon (DOC), compared to a conventional water treatment plant at 57.5% [9]. The membranes also performed exceedingly well in removing 87% and 92% of humic and building blocks, respectively, and significantly lowered the permeate chlorine demand and hazards associated with DBPs [9]. Although NF membranes are versatile in handling the NOM problem, the increasing concentration of NOM due to anthropogenic activities and adverse climate change effects, along with the diverse interactions of NOM with cations such as calcium (Ca^2+^) and magnesium (Mg^2+^) in natural water, aggravates the organic fouling problem [5]. The concentrations of Ca^2+^ and Mg^2+^ vary according to the specific region and are influenced by land type, geochemical structure, land use, biogeochemical cycles, and catchment management [10, 11]. In general context, the concentration ranges of Ca^2+^ and Mg^2+^ in surface water are between 0.5 and 2 mM [12].

Various characteristics of NOM, including its size, charge, and polarity, govern its fouling behaviour during NF. Several researchers have focused on evaluating the fouling performance of NF membranes instigated by humic acid (HA) and sodium alginate (SA) fractions of NOM [10, 13, 14]. HA represents the hydrophobic fraction of NOM, which contains large molecules rich in aromatic carbon, as well as functional groups such as carboxyl and phenols [15]. SA represents polysaccharide components with similar behaviour to the extracellular polymeric substance fraction of NOM, which are widely present in various natural waters, causing more severe fouling problems [16]. Cations such as Ca^2+^ and Mg^2+^ affect NOM properties via interactions through electrostatic charge, complex formation, and bridging with deprotonated groups of NOM and the membrane surface [3]. NOM fractions, including HA and SA, facilitate the formation of combined crystal organic aggregates, increase the gelling tendency, and act as nuclei to enhance bulk crystallization, leading to scaling and precipitation [17, 18]. The polysaccharide fraction of NOM, such as SA, has a rigid fibrous structure that forms an extensive three-dimensional crosslinked structure in the presence of Ca^2+^, significantly increasing the deposition of a dense fouling layer on the membrane surface and deteriorating NF performance [12]. Hence, there is a critical need to develop a fouling-resistant NF membrane to address this growing complex NOM issue.

Advances in membrane science and technology have resulted in the development of next-generation two-dimensional (2D) nanosheet-engineered thin-film nanocomposite (TFN) NF membranes via interfacial polymerization (IP) with enhanced organic removal and antifouling performance [19, 20]. 2D-enabled TFN NF membranes incorporated with nanosheets, such as molybdenum disulphide (MoS_2_) [21], metal carbides and nitrides (MXene) [22], and graphitic carbon nitride (g-C_3_N_4_) [23], exhibit enhanced water permeance, selectivity, and high organic fouling resistance. Among these emerging 2D nanosheets, MoS_2_ is a promising material for tailoring membranes with enhanced fouling resistance, while bridging the trade-off between permeance and selectivity [20]. MoS_2_ is a typical transition metal chalcogenide with Mo and S atoms forming hexagonal layered structures. Engineering the membrane surface with MoS_2_ nanosheets via the IP reaction resulted in careful control of the surface properties, including wettability, streaming potential, and surface morphology, imparting enhanced antifouling properties [24, 25]. In our previous study, we comprehensively evaluated the impact of OH-functionalized MoS_2_ (OH–MoS_2_) nanosheets as engineering material during the synthesis of polypiperazine amide (PPA) skin layer via the IP process [26]. The engineered membranes with an optimum concentration of 0.010 wt.% OH–MoS_2_ nanosheets resulted in 45.17% increase in pure water flux at 84.14 L m^−2^ h^−1^ against the control membrane at 57.96 L m^−2^ h^−1^, while retaining excellent salt rejection of 96.67% for Na_2_SO_4_. OH–MoS_2_-engineered membrane showed enhanced fouling resistance and organic removal during filtration studies with the HA feed solution. Although several studies have evaluated the organic fouling performance of TFN membranes, most have focused on using very high concentrations of NOM to accelerate fouling; however, this does not represent the behaviour of NOM-containing surface water [27–30]. Hence, there is a critical gap in the literature regarding the performance analysis of TFN NF membranes during filtration tests with hydrophobic and hydrophilic organic fractions at concentrations similar to natural surface water with cations, such as Ca^2+^ and Mg^2+^ [4, 25, 31].

In this study, 0.010 wt.% OH–MoS_2_ nanosheets were used to engineer PPA membrane via optimized IP reaction conditions for high organic fouling resistance. Notably, the IP reaction parameters were further optimized from our previous study to facilitate enhanced fouling resistance and NOM removal during filtration experiments with hydrophobic and hydrophilic fractions of NOM [26]. The properties to performance relationships of the engineered membranes were evaluated through extensive material characterizations. Filtration studies were conducted using lab-prepared feed solutions of HA and SA, which are synthetic surrogates of hydrophobic and hydrophilic organic fractions, respectively, with similar levels present in natural surface water. The impact of Ca^2+^ and Mg^2+^ on NOM removal and fouling performance were systematically evaluated via fouling experiments and the characterization of fouled membranes.

## Materials and methods

### Materials

Flat sheet polyethersulfone (PES) ultrafiltration (UF) membranes with a molecular weight cut-off (MWCO) of 50 kDa were procured from RisingSun Membrane Technology Co., Ltd. (China), cellulose nitrate (CN) membranes with a 0.45 µm pore size were purchased from Microanalytix Pty Ltd. (Australia), and commercial Trisep XN45 NF membranes were provided by Sterlitech. Inc., (USA). Chemicals including piperazine (PIP, ≥ 99%), trimesoylchloride (TMC, ≥ 98%), triethylamine (TEA), camphorsulfonic acid (CSA), sucrose, h-MoS_2_ powder (< 2 µm, 98%), sodium salt of HA, sodium alginate, and dialysis tubes with MWCO of 3500 Da were supplied by Sigma Aldrich (USA). n-Hexane, hydrochloric acid (32%), sodium hydroxide, calcium chloride, magnesium chloride, and sodium chloride were purchased from Merck (Australia). Deionized (DI) water was used to prepare the organic feed solutions employed in the filtration experiments.

### Synthesis of OH–MoS_2_ nanosheets

**Fig. 1:**
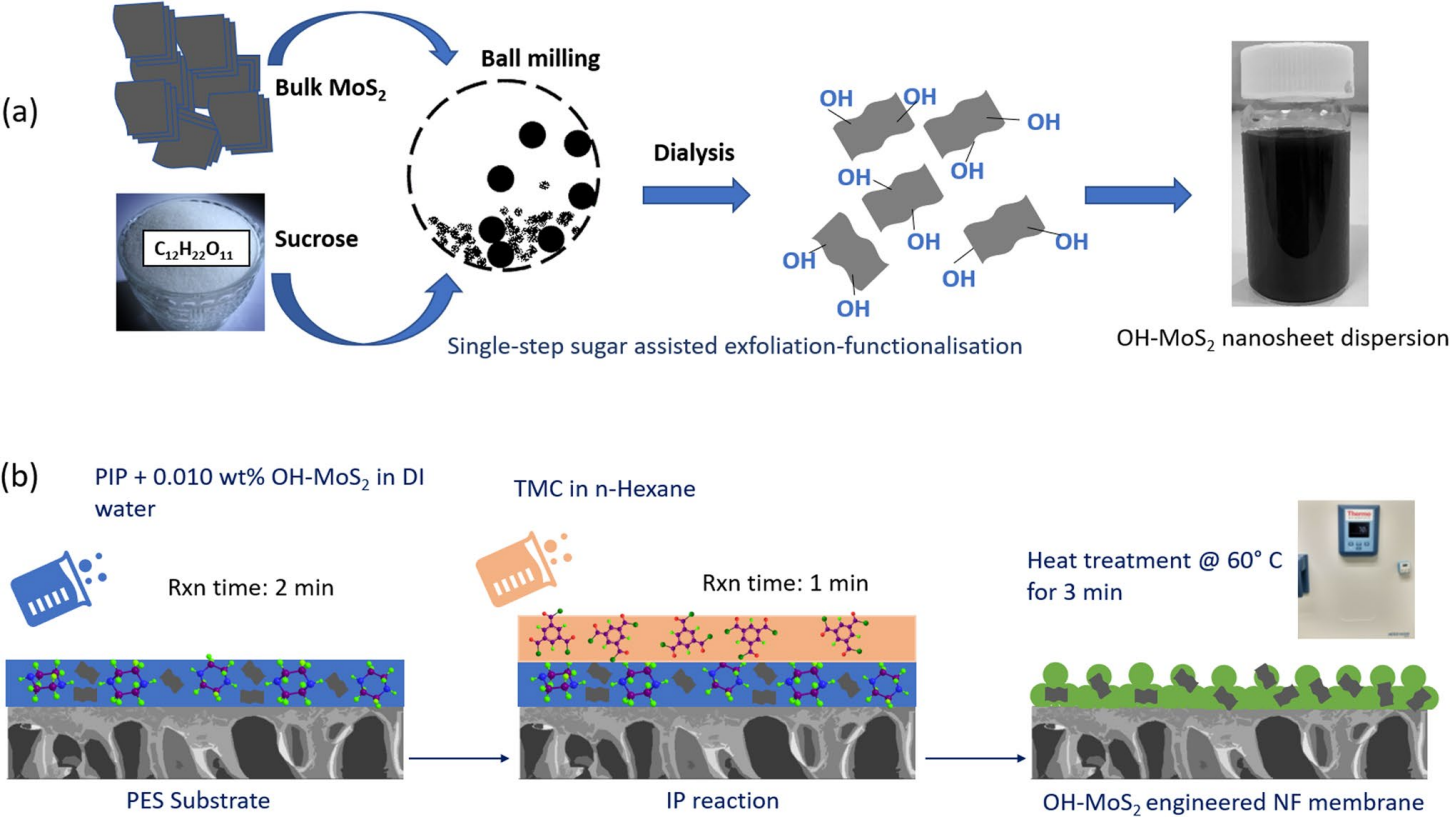
**a** Schematic representation of sucrose assisted ball milling procedure for synthesis of OH–MoS_2_ nanosheets. **b** Schematic of the TFN membrane synthesis protocol used in this study

OH–MoS_2_ nanosheets were synthesized via a simple single-step mechanochemical exfoliation using sucrose and bulk MoS_2_ powder [26, 32]. Bulk MoS_2_ powder and sucrose in a weight ratio of 1:50 were mixed in a planetary ball mill (Pulverisette 7, Fritsch) at ambient temperature under a nitrogen atmosphere. The ball milling was conducted for 3 h at 400 rpm. At the end of the ball milling process, the OH–MoS_2_ nanosheets were dispersed in ultrapure water and dialyzed for 7 days to eliminate the unreacted sucrose. Finally, hydrophilic and ultra-small OH–MoS_2_ nanosheets in water were obtained. The protocol used for the nanosheet synthesis is shown in Fig. 1a.

### Synthesis of NF membranes

2D-enabled NF membranes were synthesized via modified IP reaction method abridged from our prior study [26]. The PES support membrane was clamped in a plate-and-frame assembly prior to synthesis. The aqueous phase containing 3 wt.% PIP, 3 wt.% CSA, 3 wt.% TEA, and 0.010 wt.% OH–MoS_2_ was allowed to react with the PES support membrane or 2 min. To facilitate homogenized dispersion of nanosheets in the aqueous phase, an ultrasonic cleaner was used at 100 W for 30 min. Subsequently, a soft rubber roller was used to uniformly distribute the aqueous phase over the membrane surface. This was followed by 1 min contact with organic phase containing 0.15 wt.% of TMC in n-Hexane. The IP reaction is facilitated at the interface of the aqueous and organic monomer phases, forming a PPA-selective skin layer. After the IP reaction the organic phase was removed, and the membrane was subsequently washed with n-hexane. Finally, the membranes were exposed to heat treatment at 60 °C for 3 min in a digital oven and stored in DI water at 4 °C until use. A schematic of the IP reaction protocol used in this study is represented in Fig. 1b. Control membranes without the nanosheets were synthesized via similar IP reaction protocol without incorporation of nanosheets in the aqueous phase. The synthesized membranes are termed as control PPA and OH–MoS_2_ PPA. To benchmark the performance commercial XN 45 membranes were also tested.

### Physicochemical characterization of nanosheets and membranes

The physicochemical properties of the nanosheets and membranes were analysed with the characterization methods described below and follows the protocol from our previous experimental publication [26]. Prior to the analysis, all membrane samples were dried in a desiccator for 24 h.

The chemical composition of the nanosheets and membranes was evaluated by attenuated total reflectance-Fourier-transform infrared (ATR-FTIR) spectroscopy. For this Bruker Lumos FTIR spectrometer with wavenumbers ranging from 600 to 4000 cm^−1^ and 64 scans with a resolution of 4 cm^−1^ was employed. Atomic force microscopy (AFM) was employed using a Bruker Multimode 8 instrument operating in non-contact mode at room temperature for analysing the morphology of nanosheets and the surface roughness of the membrane. For the tests with nanosheets, the dispersion was diluted to 0.001 mg mL^−1^ and imaged on a mica substrate. Prior to testing, samples were dried at room temperature. The root average arithmetic roughness (*R*_a_) was evaluated for a scanning area of 5 µm × 5 µm for measuring the membrane surface roughness. The surface morphologies of the nanosheets and membranes were analysed using a Supra 55 VP (ZEISS, Germany) field-emission scanning electron microscope (FE-SEM) instrument. For nanosheet analysis, the nanosheet dispersion was diluted to 0.001 mg mL^−1^ and dried over a silicon substrate for imaging. The surface micromorphologies of the membranes were recorded at 5 kV and working distance of 10 mm. Both nanosheet and membrane samples were subjected to gold coating prior to imaging to improve the conductivity of the samples.

KSV Attention-Theta contact angle apparatus was employed to for the analysis of membrane surface hydrophilicity. Three random points on the membrane were chosen for the CA measurements, and the average values were reported. A Surpass 3 Anton Paar Zeta potential analyser was used to measure the streaming potential of the fabricated membranes with 1 mM NaCl solution as the background electrolyte. The zeta potentials of the nanosheet dispersion and NOM feed solution were evaluated using a Zetasizer Nano ZS (Malvern Instruments). Microscopic images of the air-dried droplets of the NOM feed solutions were obtained using a benchtop optical microscope (Olympus).

### Evaluation of NF membrane performance

The NF membrane performance were evaluated based on the experimental procedures developed from published study [26]. The separation performances of the NF membranes were evaluated by employing a Sterlitech CF042 crossflow filtration setup, exposing an active surface area of 0.0042 m^2^. First, the membrane compaction was achieved at 8 bar for 3 h, after which the pure water flux was evaluated at 6 bar for 1 h. This was followed with filtration tests using feed solution containing either HA or SA, which represent the hydrophobic and hydrophilic components of NOM, respectively. The experiments were carried out at ambient room temperature of 25 °C. To mimic the surface water conditions, the feed water concentration was maintained at 8 mg L^−1^, with a pH of 7 ± 0.05 (using 0.1 HCl or NaOH solution). To evaluate the impact of cations on membrane filtration performance, the feed water containing the organic solution was spiked with 0.5 and 1.0 mM Ca^2+^ and Mg^2+^ using CaCl_2_ and MgCl_2,_ respectively, to mimic the cation concentration in real surface water.

For all the filtration experiments, a HYDRA-CELL pump was used to pump the feed solution, and the flux was calculated using Eq. (1):

1 $$J = \frac{Q}{A \times t}$$

where *J* is the flux (L m^−2^ h^−1^), Q is the permeate volume (L), *A* is the active surface area of the membrane (m^2^), and *t* is time (h). The permeance of each membrane was calculated using Eq. (2).

2 $${\text{WP}} = \frac{J}{\Delta p}$$

where WP is the water permeance (L m^−2^ h^−1^ bar ^−1^), and $\Delta p$ is the pressure difference (bar).

DOC removal was measured using a total carbon analyser (TOC-L, SHIMADZU Corporation, Japan) for sample aliquots of the feed and permeate. The removal values were evaluated using Eq. (3):

3 $$R \left( \% \right) = 1 - \frac{{C_{{\text{p}}} }}{{C_{{\text{f}}} }}$$

where *R* is the removal (%); *C*_p_ is the DOC level in the permeate (mg L^−1^ for DOC, cm^−1^ for UV_254_, and µS cm^−1^ for salt rejection); and *C*_f_ is the DOC level in the feed (mg L^−1^ for DOC, cm^−1^ for UV_254,_ and µS cm^−1^ for salt rejection).

To evaluate the antifouling performance of the membranes, the normalized flux was calculated using Eq. (4):

4 $$N_{{\text{F}}} = \left( {\frac{{J_{{\text{T}}} }}{{J_{0} }}} \right) \times 100\%$$

where *N*_F_ is the normalized flux, *J*_T_ is the flux at the termination of the experiment, and *J*_0_ is the initial flux.

At the end 6 h fouling experiments, the membrane coupons were cleaned with pure water for 20 min, after which the relative flux recovery ratio (*R*_re_) was evaluated with fresh water according to Eq. (5):

5 $$R_{{{\text{re}}}} = \frac{{F_{{{\text{re}}}} }}{{F_{0} }} \times 100\%$$

where *F*_0_ is the initial pure water flux prior to the fouling experiments and *F*_re_ is the pure water flux after cleaning.

The salt rejection of the membranes was evaluated using feed water containing 1000 mg L^−1^ Na_2_SO_4_, MgSO_4_, CaCl_2_, and NaCl. All experiments were conducted in triplicate, and the average values were reported for the membrane performance experiments.

## Results and discussion

### Characterization of OH–MoS_2_ nanosheets

**Fig. 2:**
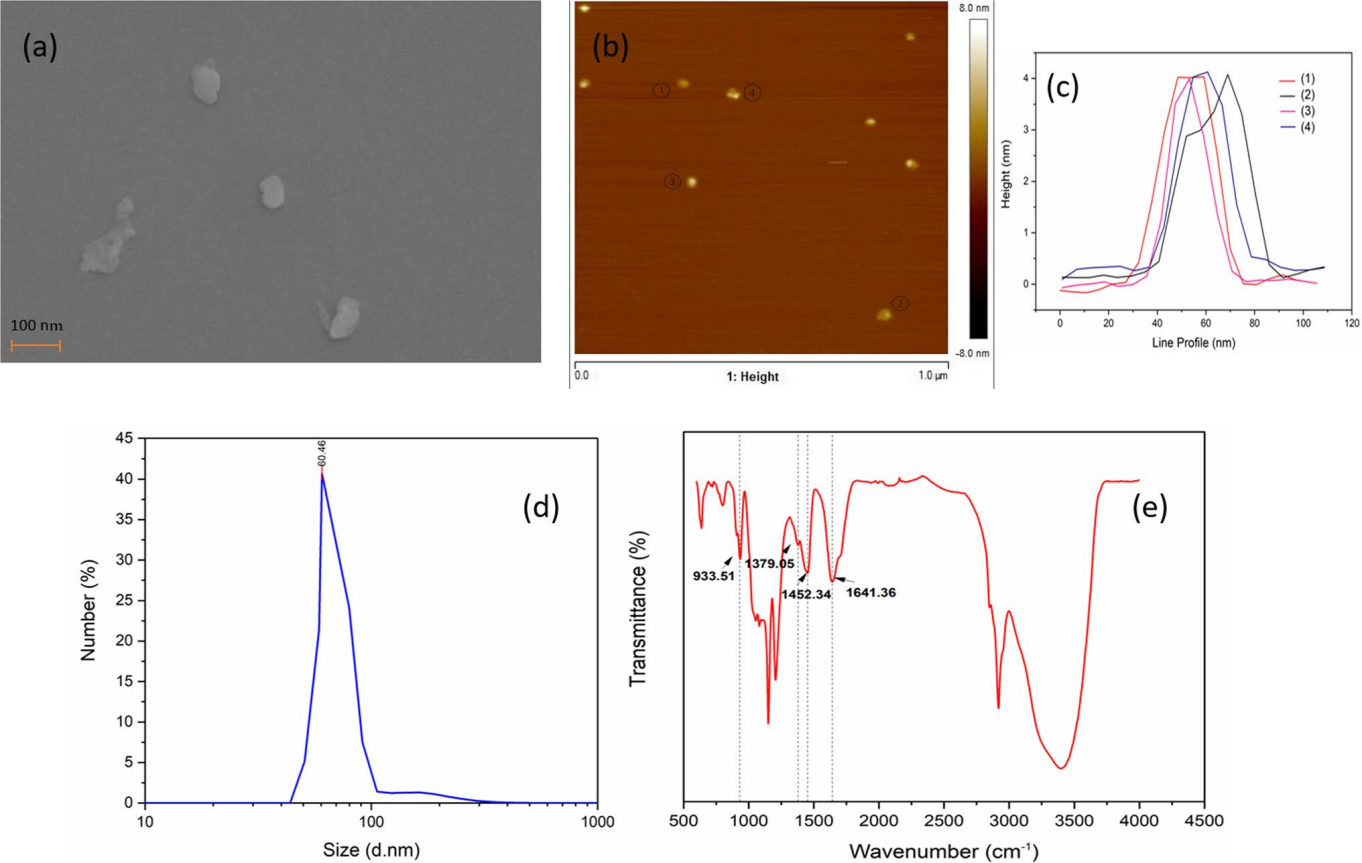
Characterization of the synthesized OH–MoS_2_ nanosheets: **a** SEM image, **b** AFM image, **c** corresponding line profiles of nanosheets, **d** average size distribution of nanosheets evaluated using Zetasizer data, and **e** FTIR spectrum of OH–MoS_2_ nanosheets coated polycarbonate membrane

The surface morphologies of the exfoliated OH–MoS_2_ nanosheets were examined using SEM and AFM. Figure 2a shows the layered structure of OH–MoS_2_ nanosheets in the size range of 65–91 nm. Additional file 1: Figure S1(a–d) presents SEM images of OH–MoS_2_ nanosheets at various magnification levels. The average size and thickness of the nanosheets observed by AFM in Fig. 2b, c were ~ 55 nm and ~ 4 nm, respectively, confirming their ultra-small size and 2D few-layered structure. Single layer of MoS_2_ nanosheet has a thickness of 0.62 nm, and hence, the synthesized OH–MoS_2_ nanosheets in this study have 6–7 layered structures. The OH–MoS_2_ nanosheet dispersion exhibited a high zeta potential of − 45.61 mV at pH 7, confirming its hydrophilic nature, while the zetasizer data as shown in Fig. 2d also revealed that the average size distribution of the nanosheets was 60.46 nm. The slight variations in nanosheet size measurements obtained from SEM, AFM, and Zetasizer are expected due to the inherent differences in measurement techniques and conditions. SEM and AFM provide surface-level and topographical insights into nanosheets, respectively, while Zetasizer measures the hydrodynamic size in a solution.

In our study, we employed OH–MoS_2_ nanosheet dispersion to coat a polycarbonate membrane, and the resulting FTIR spectrum is presented in Fig. 2e. To further substantiate the functionalization of these nanosheets, we have included the FTIR spectra of both the bulk MoS_2_ powder and the polycarbonate membrane in Additional file 1, Figure S2. Notably, for the bulk MoS_2_ analysis, we utilized the KBr method to acquire the FTIR data. In Additional file 1: Figure S2, the FTIR spectra of bulk MoS_2_ powder exhibited prominent peak around 472.54 cm^−1^ which corresponds to the characteristics Mo–S bond vibrations, while the polycarbonate membrane showed notable peaks at 1149.53 cm^−1^ and 1205.46 cm^−1^ corresponding to the symmetric and asymmetric stretching vibrations of C–O–C bond within the polycarbonate molecule [33, 34]. The FTIR spectra of OH–MoS_2_ nanosheets (Fig. 2e) present peaks at 1641.36 cm^−1^ and 1452.34 cm^−1^ which corresponds to Mo–S, while the peaks at 933.51 cm^−1^ and 1379.05 cm^−1^ represent S–S and Mo–O bond, respectively [26, 35, 36]. The broad peak ranging between 2900 and 3500 cm^−1^ is due to the symmetric stretching of the hydroxyl groups of the nanosheets [36]. The synthesized MoS_2_ nanosheets possess ultra-small size, high hydrophilicity, and abundant OH functional groups, facilitating their seamless integration as 2D nanofillers into the polymer matrix, giving rise to a new class of high-performance nanofiltration membranes [26].

### Characterization of membranes

**Fig. 3:**
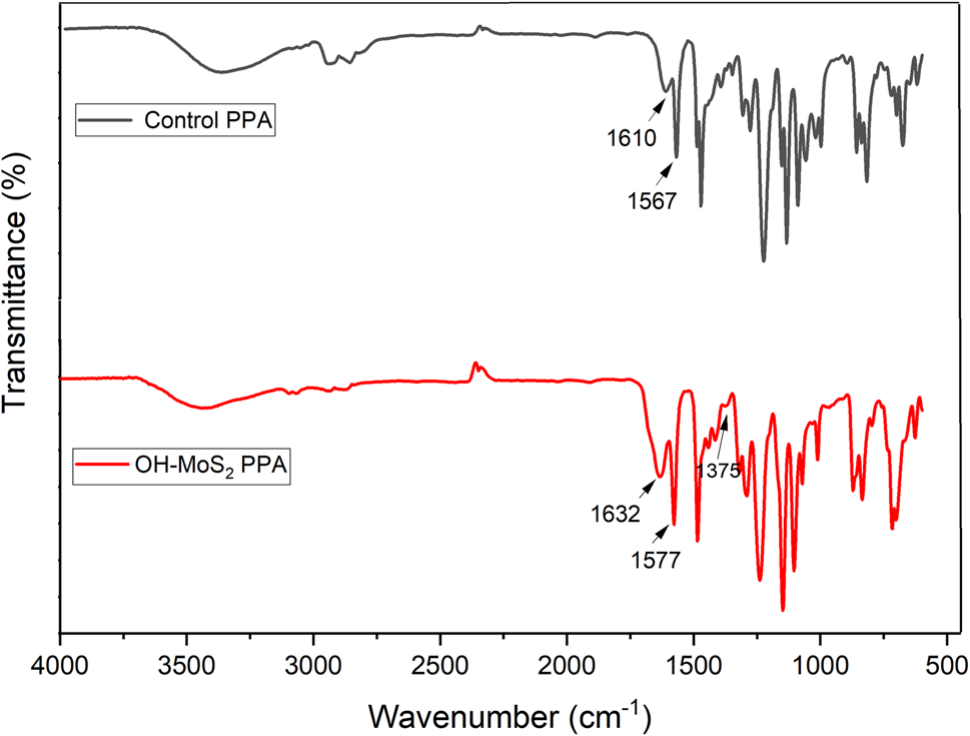
ATR-FTIR spectra of control PPA and OH–MoS_2_ PPA membranes

FTIR was employed to analyse the chemical compositions of the control PPA and TFN membranes with 0.010 wt.% OH–MoS_2_ nanosheets, and the results are shown in Fig. 3. Accordingly, for the control PPA membrane, the bands appearing at approximately 1610 cm^−1^ and 1567 cm^−1^ corresponds to the C=O stretching vibration of the amide I bond and the C–N stretching and C–N–H vibrations of the amide II band, respectively, which confirms the formation of a polyamide skin layer [37]. The OH–MoS_2_ PPA membrane exhibited a redshift of these peaks to 1632 cm^−1^ and 1577 cm^−1^ which could be ascribed to the enhanced IP reaction in the presence of OH–MoS_2_ and its interaction with the polymer matrix. The OH group grafted on the surface of the nanosheet facilitated hydrogen bonding with the PIP molecules, enhancing the IP reaction and covalent bonding with the unreacted carboxylic groups of the polymer matrix, significantly affecting amide formation [26]. The band at 3200–3600 cm^−1^ and 1485 cm^−1^ could be ascribed to the hydroxyl stretching vibration of the carboxylic groups, whereas the peak at 1570 cm^−1^ could be attributed to the stretching vibration of the benzene ring [38]. In addition, the OH–MoS_2_ PPA membrane exhibited bands at 640 cm^−1^ and 1375 cm^−1^, assigned to MoS_2_ and Mo–O bonds, respectively, confirming the successful incorporation of nanosheets within the polymer matrix [36].

**Fig. 4:**
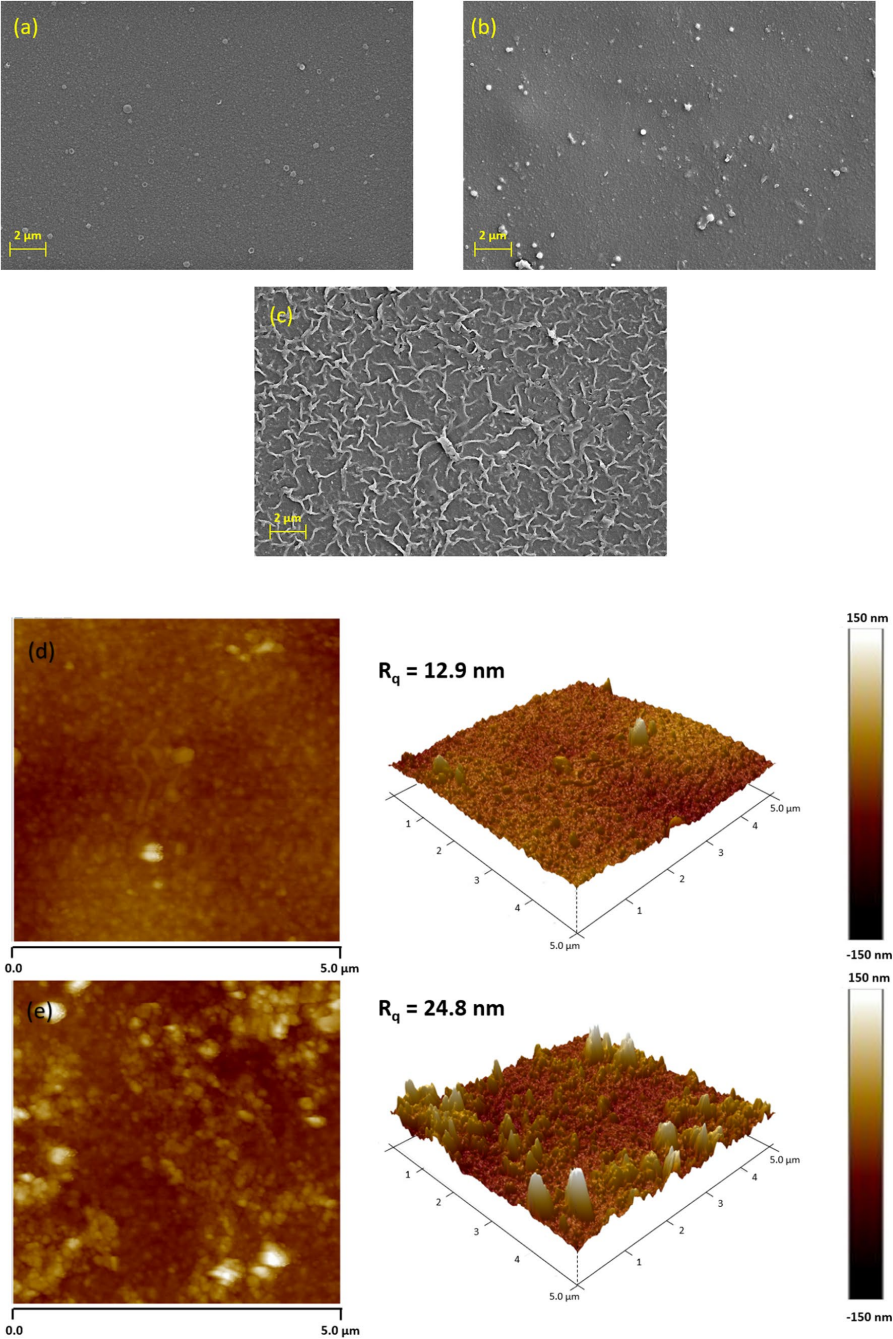
SEM surface morphologies of **a** XN 45, **b** control PPA and **c** OH–MoS_2_ PPA membrane, 2D and 3D AFM images of **d** control PPA and **e** OH–MoS_2_ PPA membrane

The surface morphologies of the membranes were examined using SEM to determine the impact of OH–MoS_2_ nanosheets on the membrane surface. As shown in Fig. 4a, XN 45 membrane exhibited a smooth surface with nodular structures. The control PPA membrane (Fig. 4b) exhibited randomly dispersed bubble-like granular structures, which could be related to the swift IP reaction between the monomers. During the IP reaction, the PIP molecules diffuse into interface of aqueous and organic phases and react with TMC, facilitating the formation of a polyamide layer [19]. This extremely thin layer serves as a seeding location to draw additional PIP molecules for polymerization. This can push PPA tufts aside and cause bubble-like nodules to grow on the surface. The local concentration of PIP in the reaction zone controls the density and number of these structures [39]. Engineering the TFN membranes with OH–MoS_2_ nanosheets at the optimum concentration of 0.010 wt.% resulted in the formation of extensively crumpled fishnet-like structures, as shown in Fig. 4c. This can be related to the local enrichment of PIP molecules via hydrogen bonding with the nanosheets, which promotes the IP reaction around the nanosheets [40].

The hydroxyl groups present on the edges of the OH–MoS_2_ nanosheets can lead to covalent interactions with TMC, improving the crosslinking degree and creating multiple growth directions, resulting in extensive fishnet-like structures [41]. Similarly, crumpled surface morphology was reported in studies incorporating modified MoS_2_ (TA-MoS_2_) nanosheets, and the change was related to the promoted IP reaction and participation of functional groups attached to the nanosheets [31]. It is noteworthy that the OH–MoS_2_ nanosheet exhibited no aggregation and unselective defect formation owing to the improved compatibility with the polymer matrix facilitated by OH functionalization of the nanosheets. Further the SEM cross-sectional image analysis revealed noteworthy differences in the average thickness of the selective polyamide layer between the control PPA and OH–MoS_2_ PPA membranes. The control PPA membrane exhibited an average thickness of 130 nm, while the OH–MoS_2_ PPA membrane displayed a slightly thicker PPA layer, measuring 206 nm. The promoted IP reaction along with the orientation of nanosheets in the vertically and multiple polymer growth directions resulted in increasing the thickness of the selective polyamide layer for the OH–MoS_2_ PPA membrane as shown in Additional file 1: Figure S3 (a-b). Despite the increased PPA layer thickness in the MoS_2_ PPA membrane, the interfacial voids and crumpled structure within the membrane surface enhanced the membrane permeance [19, 26].

AFM analysis of the membrane surface deciphered the impact of incorporating OH–MoS_2_ nanosheets into the PA layer. The 2D and 3D AFM images with a scan area of 5 µm × 5 µm for the control PPA and OH–MoS_2_ PPA are shown in Fig. 4d, e. As shown in Fig. 4d, the control PPA membrane consists of several peaks due to bubble-like nodular structure of the PPA film, as supported through SEM analysis. The surface roughness values of the membranes are listed in Table 1. The control PPA membrane exhibited a smoother surface with *R*_q_ value of 12.9 nm, while the XN 45 membrane showed moderate *R*_q_ value of 18.6 nm (Fig. 4d and Additional file 1: Figure S4). Incorporation of OH–MoS_2_ nanosheet remarkably increased the roughness *R*_q_ value to 24.9 nm due to the formation of crumpled fishnet-line structures, which is in agreement with the SEM observations in Fig. 4c. In general, a rougher membrane surface facilitates increased surface area available for filtration, which enhances membrane permeance. This leads to the formation of a water hydration layer over the membrane surface, boosting its antifouling performance [42]. The increased surface roughness was attributed to the interaction of OH–MoS_2_ nanosheets during the IP reaction, leading to a local enrichment of PIP molecules in the organic phase, improving the reaction kinetics, and facilitating multiple growth directions with visible fishnet-like protuberances [31].

**Table 1:** Tabulation of membrane surface properties of membranes tested in this study

Membrane ID	Roughness (*R*_q_ in nm)	CA (°)	Streaming potential at pH 7 (mV)
XN 45	18.6 ± 1.81	39 ± 1.24	− 35.46
Control PPA	12.9 ± 1.81	43 ± 1.12	− 28.46
OH–MoS_2_ PPA	24.9 ± 1.12	29.20 ± 0.86	− 44.45

The hydrophilicity of the membranes was measured using CA analysis. Hydrophilic surface is linked with lower CA, which enhances membrane wettability and improves the fouling resistance of the membrane [43]. A hydrophilic surface prevents the deposition of NOM onto the membrane and facilitates higher removal of NOM due to the formation of water hydration layer [5]. Therefore, engineering membrane surface hydrophilicity and roughness is key to developing organic fouling-resistant NF membranes [7]. The roughness data and CA measurements are tabulated in Table 1. The OH–MoS_2_ PPA membrane with a CA of 29.20° exhibited hydrophilic surface than the control PPA and XN 45 membrane with a CA of 43° and 39°, respectively. This was due to the hydrophilic characteristics of the incorporated 2D OH–MoS_2_ nanosheets and the ability of the hydroxyl groups of the nanosheets to absorb water molecules via hydrogen bonding [32]. Nevertheless, the enhanced hydrophilicity of the membrane surface can act as a double-edged sword during filtration with hydrophilic fractions of NOM [5]. In this study, both hydrophilic and hydrophobic NOM were used to evaluate organic fouling and NOM removal performance. The enhanced membrane hydrophilicity can boost the formation of a water hydration layer, mitigating the deposition of hydrophobic organic surrogates such as HA; however, it can increase the bridging and adsorption of hydrophilic NOM fractions such as SA and bovine serum albumin (BSA), which can aggravate fouling [4]. Therefore, investigating membrane surface hydrophilicity and its interaction with NOM is highly relevant for engineering fouling-resistant membranes.

In addition to membrane surface hydrophilicity, the surface charge also controls the removal of NOM and fouling performance [5]. The surface charge properties of control PPA and OH–MoS_2_ PPA were evaluated using streaming potential measurements. In general, PPA membranes possess a negative charge at neutral pH due to the disassociation of the free carboxylic groups present in the polymer chain. The IP reaction gives rise to a polyamide structure; however, the residual unreacted acyl chloride groups hydrolyse into carboxylic groups, which deprotonate at pH values higher than their pKa [44]. Membranes with negative surface charges are always preferred for NOM removal, as the NOM moieties possess a negative charge at neutral pH, enhancing their removal and fouling mitigation properties [5]. The electrostatic interactions between NOM and the membrane surface restrict the deposition of organic matter on the membrane surface, thereby alleviating membrane fouling. The OH–MoS_2_ PPA membrane possesses a negative charge of − 44.45 mV which is almost 56% higher than the control PPA at − 28.46 mV and 25% higher than XN 45 membrane at 35.46 mV as shown in Table 1. This was due to the electronegative nature of the OH–MoS_2_ nanosheets on the membrane surface [32]. The hydroxyl groups attached to the nanosheets also increase the negative charge of the membrane [32]. As the NF membrane separation is also dependent on the electrostatic interactions between the solutes and the membrane surface, the enhanced surface negative charge on the incorporation of OH–MoS_2_ nanosheets could facilitate increased repulsive behaviour towards negatively charged salt ions and NOM molecules, which further improves the selectivity and fouling resistance of the membranes [25].

### Filtration performance of NF membranes

#### Pure water permeance and salt rejection

**Fig. 5:**
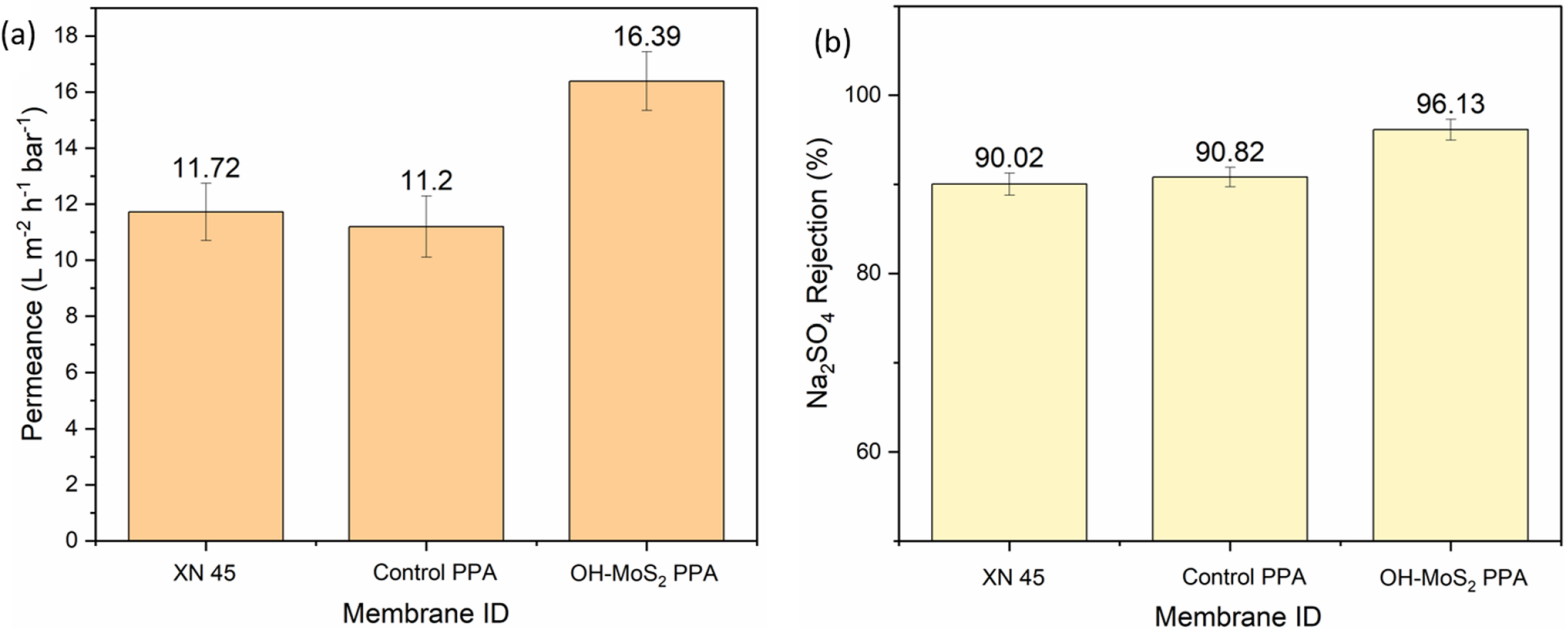
**a** Pure water permeance and **b** Na_2_SO_4_ rejection for the NF membrane evaluated in this study

TFN NF membranes incorporated with 0.010 wt.% OH–MoS_2_ nanosheets in the aqueous phase during the IP reaction enhanced the pure water permeance by 46.33% from 11.20 to 16.39 L m^−2^ h^−1^ bar^−1^ against the control PPA membrane, as depicted in Fig. 5a. The TFN membrane also exhibited excellent salt rejection performance, bridging the trade-off between enhanced water permeance and rejection. This was mainly attributed to the enhanced electronegative properties of the membrane surface with OH–MoS_2_ nanosheets [45]. Studies have also reported that partial coverage of membrane pores by MoS_2_ nanosheets can increase the salt separation properties owing to the additional sieving effect [37]. The TFN membrane exhibited salt rejection (Na_2_SO_4_) of 96.3% compared with 90.82% for the control membrane, as shown in Fig. 5b. The TFN membrane incorporated with OH–MoS_2_ nanosheets showed higher water flux and salt rejection compared with XN 45 NF membrane, which supports its relevance and practical application for surface water treatment. The modified TFN membrane also exhibited tremendous increase in rejection performance for MgSO_4_ and NaCl at (85.2% → 94.16%) and (36.48% → 70.45%), respectively, compared to the control PPA membrane. This performance enhancement was linked to the OH–MoS_2_ nanosheets and its ability to engineer membrane surfaces with suitable hydrophilic and electronegative properties. The hydrophilic properties of OH–MoS_2_ nanosheets could be favourable to form hydrogen bonds with water molecules, facilitating ultrafast transport, and enhancing membrane water permeance [25]. The hydrophilicity and surface charge of the TFN membrane surface increased upon incorporation of the nanosheets, as evidenced by the CA and zeta potential values in Table 1. The surface micromorphology was transformed into rough crumpled fishnet-like structures owing to the interactions of the OH–MoS_2_ nanosheets during the IP reaction via hydrogen bonding. This leads to the formation of additional selective nanovoids and micropores, which enhance the flux of the membranes [31].

The presence of Mo–O and S=O bonds in the OH–MoS_2_ nanosheets promotes the IP reaction and enhances the degree of crosslinking of the polymer matrix, improving the selectivity of the membrane [46]. The intrinsic nanopores of OH–MoS_2_ nanosheets, along with their interfacial and interlayer voids, can also contribute to improving water permeance [32]. Comparison with existing studies on various 2D engineered TFN NF membranes incorporating MoS_2_, BN, GO, and g-C_3_N_4_ indicated that the OH–MoS_2_ membrane in this study exhibited superior performance with water permeance and salt rejection at 16.39 L m^−2^ h^−1^ bar^−1^ and 96.3%, respectively, bridging the trade-off between permeance and rejection, as shown in Additional file 1: Table S1. Furthermore, the modified TFN NF membrane in this study had approximately twice the water permeance of previous studies that used MoS_2_ for TFN membrane synthesis [24, 25, 46]. This enhanced performance supports a new strategy for engineering membranes with OH–MoS_2_ nanosheets for emerging nanofiltration applications.

### Fouling resistance performance and NOM removal

**Fig. 6:**
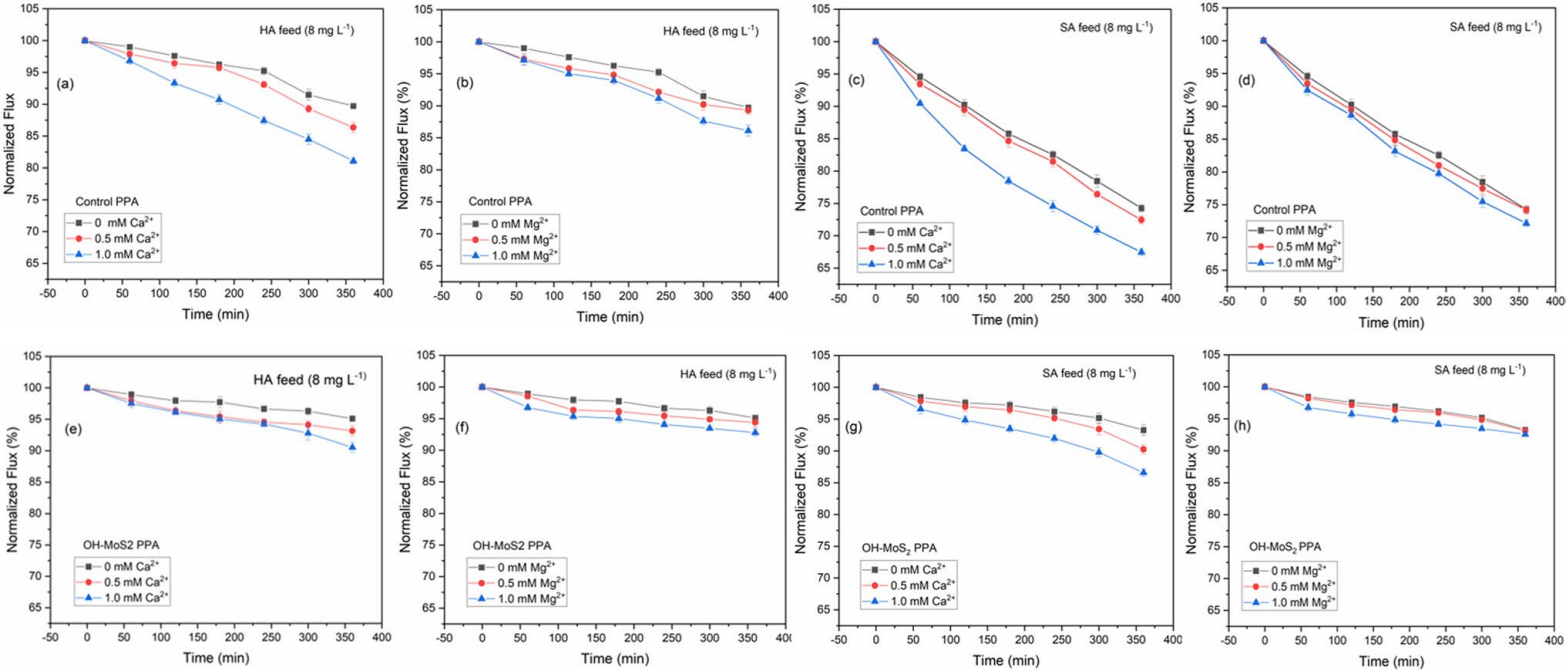
Trends of normalized flux during 6 h fouling tests conducted under different divalent cation contents and NOM feed solutions for the control PPA (**a**–**d**) and OH–MoS_2_ PPA (**e**–**h**)

**Fig. 7:**
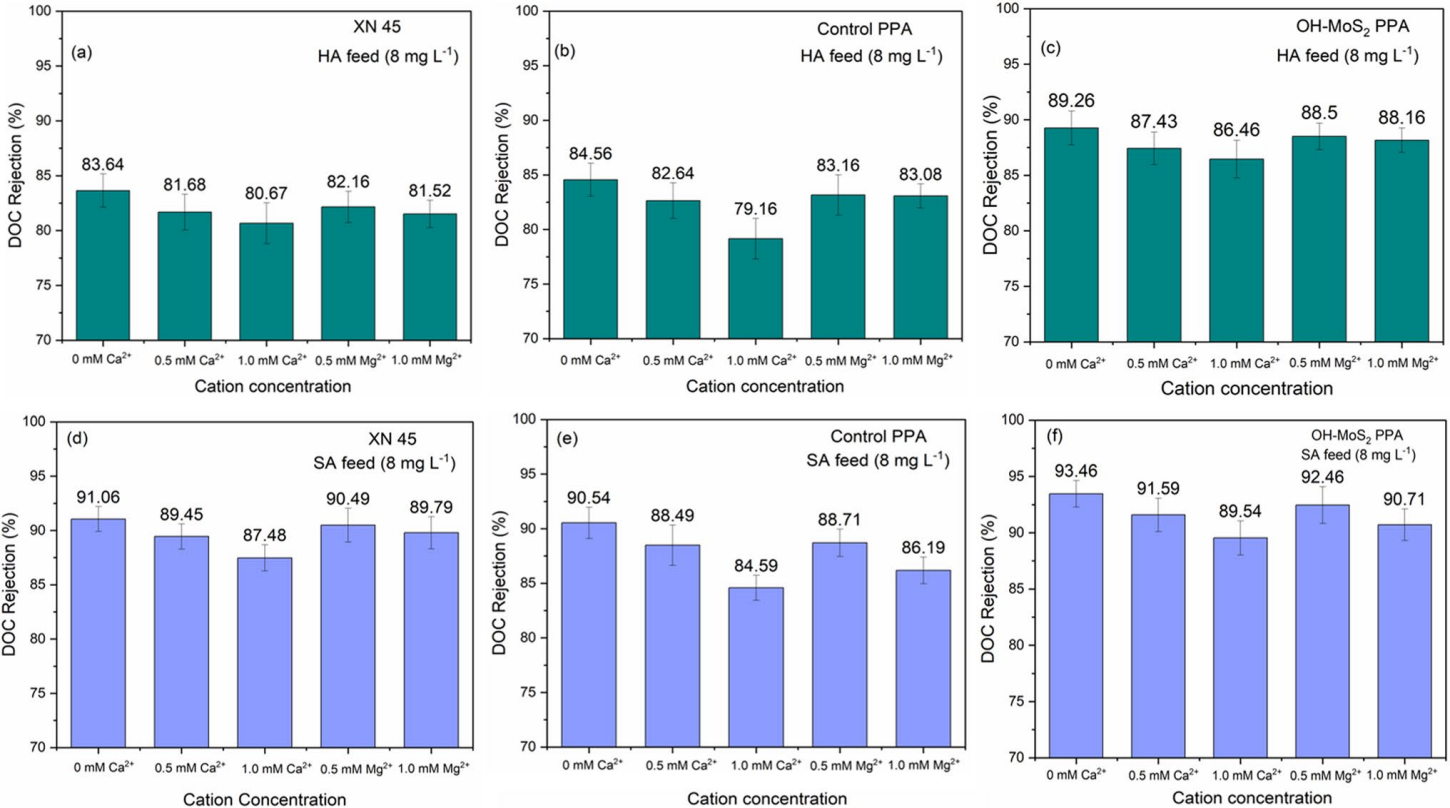
NOM rejection in terms of DOC during filtration studies with HA feed solution for **a** XN45, **b** control PPA, and **c** OH–MoS_2_ PPA, and with SA feed solution for **d** XN45, **e** control PPA, and **f** OH–MoS_2_ PPA

Analysis of the fouling experiments and normalized flux profiles showed that the OH–MoS_2_ nanosheets-engineered TFN membrane resulted in enhanced fouling resistance and NOM removal for both hydrophobic and hydrophilic NOM fractions. Fouling experiments with the control PPA membrane exhibited normalized fluxes of 89.71% and 74.25% at the conclusion of the 6 h tests for HA and SA NOM fractions, respectively (Fig. 6a–d), while the OH–MoS_2_ PPA membrane showed a normalized flux of 95.09% and 93.26%, respectively (Fig. 6e–h). To benchmark the fouling trend, commercial XN 45 membranes were also tested under similar conditions and the normalized flux profiles are include in Additional file 1: Figure S5 (a–d). At the end of 6 h fouling experiments, the normalized flux was at 88.79% and 78.54% for HA and SA feed solutions, respectively. The commercial XN 45 membrane showed rejection of 83.64% and 91.06% for HA and SA in terms DOC, respectively, as shown in Fig. 7a, d. The OH–MoS_2_ PPA membrane exhibited enhanced NOM removal at 89.26% and 93.46% for HA and SA feed solutions, respectively, while the control PPA NOM removal was 84.56% and 90.54%, respectively, as shown in Fig. 7c, f and Fig. 7b, e. The improved performance of the OH–MoS_2_ PPA membrane can be attributed to its enhanced hydrophilicity, electrostatic repulsion, and modified surface morphology [24]. The higher negative charge properties of the OH–MoS_2_ PPA membrane resulted in effective repulsion of NOM molecules. Both HA and SA molecules hold a negative charge under neutral pH maintained for the feed solution, which enhances the repulsion between NOM and the negatively charged membrane surface [5]. The increased surface roughness and hydrophilicity of the membrane, as evidenced through AFM and CA analysis, can support the formation of a water hydration layer reducing the attachment of NOM to the membrane, improving both the antifouling and NOM removal performance [47]. The literature also suggests that a super-hydrophilic membrane surface can worsen fouling owing to its interactions and the deposition of hydrophilic NOM fractions [5]. However, the OH–MoS_2_ PPA membrane performed well and exhibited excellent performance when handling both the SA and HA feed solutions, establishing its operational versatility in handling both the hydrophobic and hydrophilic fractions of NOM. This can be attributed mainly to the electrostatic repulsive separation mechanism of NOM, which prevents the deposition of SA on the membrane surface [48]. Notably, the OH–MoS_2_ PPA membrane exhibited better performance than the commercial XN 45 membrane in terms of NOM removal and normalized flux under similar filtration conditions, as shown in Additional file 1: Table S2. In addition to membrane properties such as hydrophilic and electrostatic interactions, membrane surface roughness significantly impacts the fouling behaviour of NF membranes [7]. The enhanced organic fouling and removal performance of OH–MoS_2_ PPA could also be attributed to the increased surface roughness on incorporation of the nanosheet. Heightened surface roughness amplifies the available filtration area and facilitates the creation of a hydration layer on the membrane’s surface which pushes away the organic molecules reducing their deposition while improving the water permeance and organic removal. Similar property to performance relationship between increased roughness and improved fouling performance has been reported for nanomaterial-engineered TFN membranes [5, 49]. While increased surface roughness can enhance water permeance and organic fouling resistance, it introduces a significant concern related to fouling entrapment. The intricate ridge and valley structure of rough membranes can trap organic foulants, leading to the severe organic fouling [7]. However, the increased hydrophilicity, negative charge, and surface roughness together improved the fouling performance for the OH–MoS_2_ PPA membrane.

The antifouling performance of the 0.010 wt.% OH–MoS_2_-enabled NF membrane was critically compared with other 2D nanosheets-enabled TFN membranes, and the results are summarized in Table 2. Among these studies, OH–MoS_2_-incorporated TFN membrane outperformed other TFN membranes, establishing its superior fouling mitigation properties for handling both the hydrophobic and hydrophilic fractions of NOM. The higher water permeance, enhanced salt rejection, and NOM removal performance, as summarized in Additional file 1: Tables S1 and S2, support the application of the OH–MoS_2_ TFN membrane for surface water treatment while exhibiting excellent fouling mitigation and NOM removal performance.

**Table 2:** Comparison of NOM fouling behaviour of OH–MoS_2_-incorporated membrane with functionalized MoS_2_ and other 2D nanosheet-engineered TFN membrane

2D nanosheet	Fouling agent	Filtration conditions	Normalized flux (%)	References
OH–MoS_2_	HA	6 bar, 360 min	95.09	This work
OH–MoS_2_	SA	6 bar, 360 min	93.26	This work
S-MoS_2_	BSA	4 bar, 60 min	91	[24]
O-MoS_2_	BSA	3.5 bar, 90 min	78	[25]
TA-MoS_2_	BSA	6 bar, 240 min	60	[29]
BN(NH_2_)	SA	6 bar, 360 min	95	[37]
MXene	BSA	16 bar, 360 min	89.9	[47]

### Impact of Ca^2+^ and Mg^2+^ on NOM fouling and removal performance

The NOM feed solution containing HA and SA (pH maintained at 7) was spiked with Ca^2+^ and Mg^2+^ (0, 0.5, and 1.0 mM) to evaluate the effects of divalent cations on NOM fouling and removal. Figure 6a–h shows the normalized flux trend of the NF membranes during the 6 h fouling experiments. All membranes employed in this study exhibited flux decline due to membrane fouling, and the presence of Ca^2+^ and Mg^2+^ worsened fouling and reduced NOM removal performance.

In the case of the HA feed solution, an increase in the Ca^2+^ concentration from 0 to 1.0 mM led to a significant decline in the normalized flux from 89.71 to 81.08% for the control PPA membrane (Fig. 6a), while the normalized flux declined from 88.79 to 80.38% for the commercial XN 45 membrane (Additional file 1: Figure S5 (a)). The OH–MoS_2_ PPA membrane still performed better than the control PPA and XN 45 membrane, with a decline in the normalized flux from 95.09 to 90.52% owing to its enhanced membrane surface properties. When the concentration of Mg^2+^ was increased from 0 to 1.0 mM, the normalized flux decreased to 86.11% and 84.74% for control PPA (Fig. 6b) and XN 45 membrane (Additional file 1: Figure S5 (b)), respectively, while it was maintained at 92.79% for OH–MoS_2_ PPA membrane. The NOM removal performances of the control PPA and OH–MoS_2_ PPA membranes are shown in Fig. 7a–c and benchmarked against the commercial XN 45 membrane. In the absence of cations, the DOC rejection of the membranes was 83.64%, 84.56%, and 89.26% for the XN 45, control PPA, and OH–MoS_2_ PPA membranes, respectively. At 1 mM concentration of Ca^2+^ ions in the feed, the DOC rejection decreased to 80.67%, 79.16%, and 86.46%, respectively, for XN 45, control PPA, and OH–MoS_2_ PPA membranes. The modified membrane exhibited excellent performance in retaining NOM rejection at detrimental Ca^2+^ levels. The DOC rejection values for XN 45, control PPA, and OH–MoS_2_ PPA membranes in the presence of 1.0 mM Mg^2+^ were at 81.52%, 83.08%, and 88.16, respectively. Ca^2+^ and Mg^2+^ significantly impact the HA properties owing to their interactions through electrostatic charge effects and complex formation and facilitate the formation of chemical bridges between HA and polymer matrix of the membrane surface [50]. Ca^2+^ and Mg^2+^ facilitated the attachment of HA to membrane surface owing to decreased interchain electrostatic repulsion between HA, and between the HA and polyamide layers [51]. Compared to Mg^2+^, Ca^2+^ exacerbated fouling and NOM removal, which could be attributed to its greater charge neutralization capability and favourable ability to attach to HA molecules [52]. This could lead to the formation of colloidal aggregates of HA with a lower zeta potential, owing to the neutralization of the negative charge caused by the favourable specific charge and complexation ability of cations. The lower impact of Mg^2+^ ions on HA fouling could also be linked to the high affinity of Mg^2+^ ions for water molecules and its strongly held hydration shell, which weakens its interaction with HA compared with Ca^2+^ [53]. In contrast to many studies reporting a higher fouling impact of HA foulants in the presence of Ca^2+^, studies have also reported a superior charge screening effect and charge density of Mg^2+^ to cause more severe fouling compared to Ca^2+^ [3, 54]. However, cation interactions with NOM and associated fouling are highly dependent on NOM content, feed water chemistry, membrane surface properties, and operating parameters of nanofiltration [5].

In addition, the presence of cations such as Ca^2+^ and Mg^2+^ in natural water promotes the formation of DBPs during the disinfection process [55, 56]. Interestingly, studies have indicated that DBP formation tends to be more pronounced in the presence of Ca^2+^ when compared to Mg^2+^ [57]. This phenomenon can be attributed to the binding of these cations to the carboxylate groups within NOM molecules, forming metal carboxylate complexes that facilitate DBP formation. The higher Lewis acidity of Mg^2+^, relative to Ca^2+^, has been identified as a key influencing parameter that enhances DBP formation. Notably, various NOM fractions, including polyols, citric acid, and humic acid, have shown an increased propensity for DBP formation in the presence of cations, as compared to substances such as resorcinol, histidine, and dicarboxylic acids [55, 57].

**Fig. 8:**
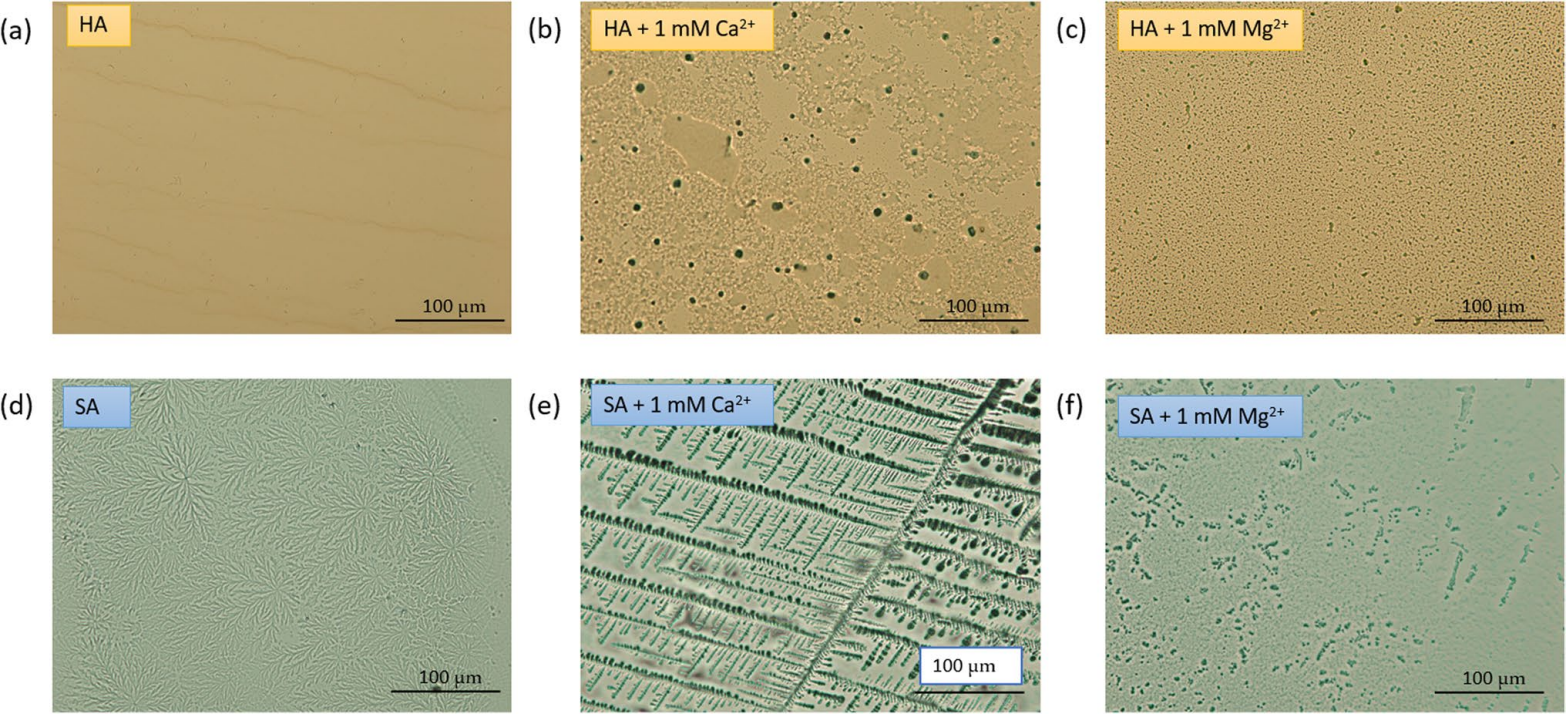
Microscopic images showing microstructures of the air-dried droplets of **a** HA solution, **b** HA + 1 mM Ca^2+^, **c** HA + 1 mM Mg^2+^, **d** SA solution, **e** SA + 1 mM Ca^2+^, **f** SA + 1 mM Mg^2+^ (Feed concentration of HA and SA solution was maintained at 8 mg L^−1^)

**Fig. 9:**
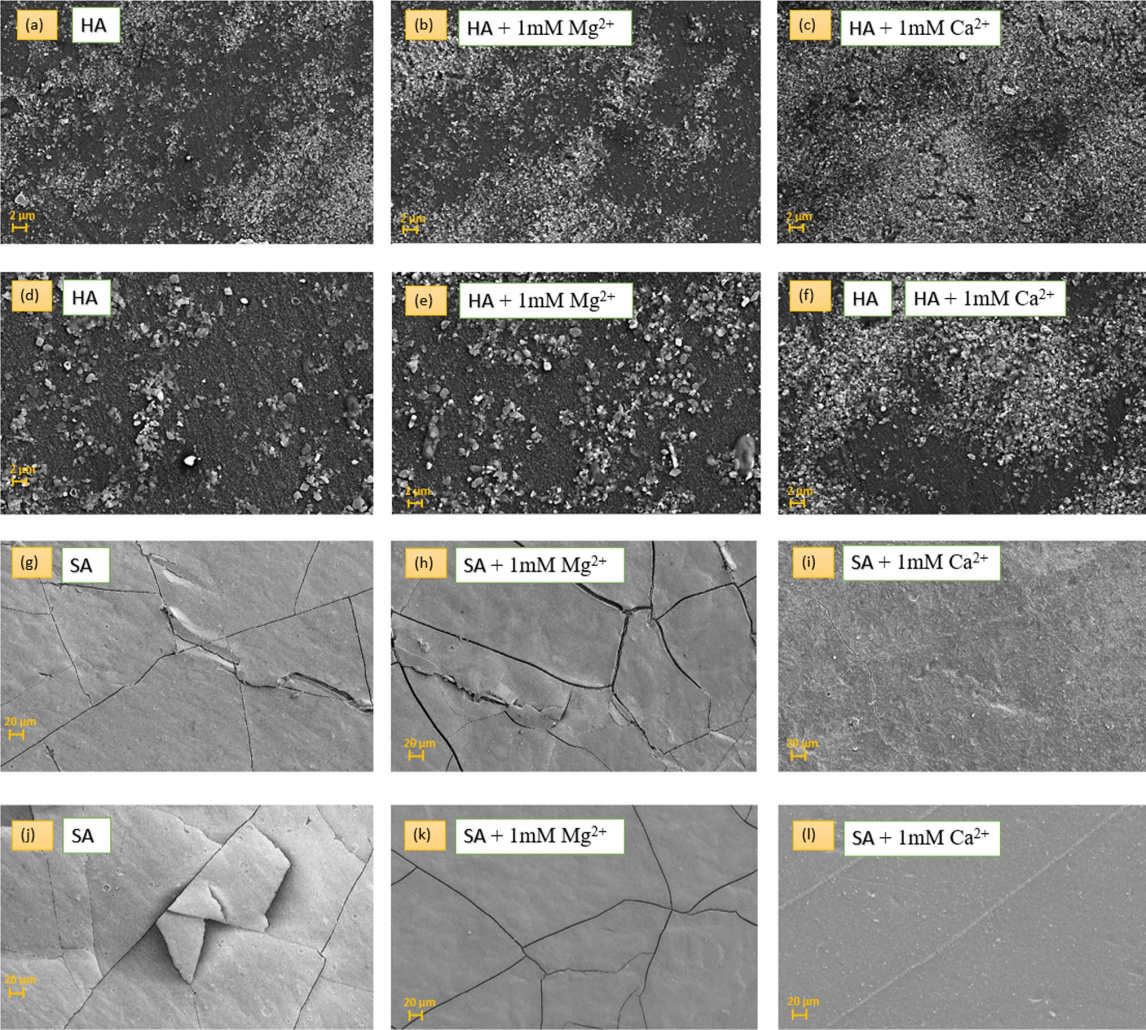
SEM images of fouled control PPA membrane with **a** HA, **b** HA + 1 mM Mg^2+^, **c** HA + 1 mM Ca^2+^, OH–MoS_2_ PPA membrane fouled with **d** HA, **e** HA + 1 mM Mg^2+^, **f** HA + 1 mM Ca^2+^, control PPA membrane with **g** SA, **h** SA + 1 mM Mg^2+^, **i** SA + 1 mM Ca^2+^, and OH–MoS_2_ membrane fouled with **j** SA, **k** SA + 1 mM Mg^2+^, **l** SA + 1 mM Ca^2+^

Figure 8a–c shows the microstructures of the air-dried droplets of the HA solution viewed under a microscope. It is evident that in the presence of 1 mM Ca^2+^ and Mg^2+^, HA molecules tend to aggregate, with Ca^2+^ causing the formation of larger aggregates. This behaviour can be linked to the neutralization of the negative charge of HA in the presence of cations, which facilitates the formation of aggregates. With increasing cation content, the negative charge of the feed solution decreased, and Ca^2+^ ions exhibited a more powerful ability to neutralize the negative charge owing to the strong affinity for carboxylic groups, as observed in Additional file 1: Table S3. SEM images of the fouled membranes after 6 h of fouling experiments were compared to understand HA fouling deposition on the membrane surface. Figure 9a–c shows the fouled control PPA membranes with 0 and 1 mM Mg^2+^ and 1 mM Ca^2+^, respectively. It can be observed that the intensity of foulant deposition increases in the presence of cations, causing a decline in flux, as reported in this study. This was mainly attributed to the reduced electrostatic repulsion between the membrane surface and HA molecules with divalent cations, facilitating its attachment on the membrane surface. The formation of large unstable HA colloidal aggregates causes pore blockage within the membrane pores, which causes significant fouling and flux decline [52]. The SEM image of fouled XN 45 membrane with HA + 1 mM Ca^2+^ as shown in Additional file 1: Figure S6 (a) also showcased significant foulant deposition. In contrast, our OH–MoS_2_ PPA membrane exhibited improved resistance to fouling compared to the commercial XN 45 membrane. The OH–MoS_2_ PPA membrane with its enhanced surface charge and hydrophilic properties lowered the deposition of hydrophobic HA compared to that of the control PPA and XN 45 membrane, as shown in Fig. 9d–e. The modified membrane’s improved hydrophilicity and surface roughness may make it easier for a water hydration layer, reducing the deposition of unstable NOM aggregates [58]. The higher electronegative charge of the engineered membrane compared to the control PPA can repel HA molecules, reducing foulant deposition [20]. Flux recoverability tests revealed that the OH–MoS_2_ PPA membrane exhibited a high relative flux recovery ratio (FRA) during filtration and cleaning tests with the HA feed, as shown in Additional file 1: Table S4. The control PPA membrane exhibited a declining trend in FRA at 92.25%, 81.54%, and 88.46% for HA, HA + 1 mM Ca^2+^, and HA + 1 mM Mg^2+^, respectively. This shows that at high Ca^2+^ levels, membrane fouling recoverability decreases because of the deposition of foulants with strong bridging between Ca^2+^, HA, and the membrane surface [59–61]. Owing to its enhanced surface properties, OH–MoS_2_ PPA membrane exhibited superior and stable FRA > 95% during all the fouling cleaning trials with varying cation content in the feed water.

Filtration tests with the SA solution exhibited worse fouling than HA. SA is a typical polysaccharide fraction of NOM that exhibits a high fouling propensity, which could be attributed to the gelling tendency of SA and its ability to form a dense fouling layer, causing a steep flux decline [62]. The normalized flux of the control PPA membrane decreased to 72.45% and 67.46% from 74.25% for feed water containing 0.5 and 1.0 mM of Ca^2+^, respectively, while OH–MoS_2_ PPA membrane retained excellent performance with normalized flux of 90.25% and 86.58% from 93.26%, respectively, as shown in Fig. 6d, h. In the case of XN 45 membrane addition of 1.0 mM of Ca^2+^ to the SA solution reduced the normalized flux from 78.54 to 70.16% as shown in Additional file 1: Figure S5 (c). The SA feed solution with 1 mM Mg^2+^ led to a slight decline in the normalized flux to 72.16% and 74.51% for the control PPA and XN 45 (Additional file 1: Figure S5 (d)) membrane, respectively, whereas the OH–MoS_2_ PPA membrane exhibited a negligible decline in the normalized flux to 92.58%. NOM rejection in terms of DOC also showed a decreasing trend with increasing Ca^2+^ and Mg^2+^. At 1 a concentration of Ca^2+^, the DOC rejection decreased from 90.54 to 84.59%, whereas at similar concentrations of Mg^2+^, DOC rejection decreased to 86.19% for control PPA membrane. OH–MoS_2_ PPA membrane maintained stable DOC rejection > 89% under varying cation concentrations, exhibiting its enhanced NOM removal performance.

Figure 9g–l shows the SEM images of fouled control PPA and OH–MoS_2_ PPA membranes tested with SA solution adding 0, 1 mM Mg^2+^ and 1 mM Ca^2+^, respectively. Increasing the Ca^2+^ led to the formation of extensive polysaccharide-crosslinked structures, which significantly increased the deposition of a dense fouling layer on the membrane surface, as depicted in Fig. 9g–l. There is a significant formation of a dense fouling layer caused by the gelling tendency of SA in the presence of Ca^2+^ and Mg^2+^. According to the Derjaguin–Landau–Verwey–Overbeek (DLVO) theory, the presence of Ca^2+^ leads to the formation of aggregates and destabilizes NOM colloids due to the electrical double layer compression of NOM, decreasing its electrostatic repulsive forces [9]. This led to the formation of SA aggregates in a thick and dense gel layer, as shown in Fig. 9i. Similarly thick and dense fouling layer was observed for XN 45 membrane fouled with SA + 1 mM Ca^2+^ feed solution (Additional file 1: Figure S6 (b)). Studies have also reported that Mg^2+^ has a negative impact on membrane fouling, as Mg^2+^ promotes the crosslinking of alginate molecules increasing its gelling tendency [62, 63]. The higher charge density of Mg^2+^ compared to that of Ca^2+^ could evoke a stronger electrostatic shielding effect, increasing SA gelling and deposition on the membrane surface. In contrast to Ca^2+^-induced gels, gelation has been shown to occur at a much greater concentration for Mg^2+^ [62, 64]. It is worth noting that this particular study focused on mimicking the surface water conditions, and hence, at similar concentrations, 1 mM Ca^2+^ induced higher fouling than Mg^2+^. The SEM images of the fouled control PPA membrane and OH–MoS_2_ PPA membrane, as shown in Fig. 9h, k at 1 mM Mg^2+^ concentration, exhibit a more loosely placed gel layer with lower intensity and packing compared to fouling with 1 mM Ca^2+^, as depicted in Fig. 9i, l.

Figure 8d–f shows the microstructures of the air-dried droplets of the SA solution viewed under a microscope. In the presence of Ca^2+^, the SA molecules form intensive combined crystal aggregates, which causes dense fouling layer development, as observed in the microscopic image of the NOM droplet (Fig. 8e) and the fouled membrane, as shown in Fig. 9i. Additional file 1: Table S3 also confirms that the zeta potential of the SA solution was lowest in the presence of 1 mM Ca^2+^. SA has a rigid fibrous structure that forms an intensively crosslinked structure in the presence of Ca^2+^, significantly worsening fouling and leading to a lower normalized flux. Due to this, the foulant layer was less responsive to water flushing, showing a declining trend for control PPA membrane with FRA at 95.46%, 80.46%, and 85.46% for SA, SA + 1 mM Ca^2+^, and SA + 1 mM Mg^2+^, respectively, whereas OH–MoS_2_ PPA membrane exhibited excellent performance with FRA > 95%. Owing to the surface charge and hydrophilicity of the OH–MoS_2_ nanosheet, the TFN membrane performed well under detrimental levels of Ca^2+^ and Mg^2+^ cations during fouling and NOM removal. Additional file 1: Table S5 summarizes the findings of the current investigation and the literature on the impact of Ca^2+^ and Mg^2+^ ions on the organic fouling of membranes. The presence of cations changes the characteristics of NOM, resulting in severe fouling of the membrane surface. Ca^2+^ induces more intense fouling than Mg^2+^. For the control PPA membrane, the hydrophilic NOM fraction of SA induced more severe fouling than the hydrophobic NOM fraction of HA did. The OH–MoS_2_ nanosheet-engineered NF membrane demonstrated excellent antifouling and NOM removal performance during filtration tests with both hydrophilic and hydrophobic fractions of NOM, as well as at detrimental levels of cations owing to its modified hydrophilicity, negative charge, and membrane surface morphology. As a result, this study demonstrates the operational adaptability of OH–MoS_2_-engineered membranes in dealing with NOM and divalent cations during surface water treatment for potable water production.

While our study highlights the promising performance of 2D MoS_2_-engineered NF membranes in addressing organic fouling and NOM removal, it is imperative to acknowledge the practical constraints when considering their scale-up for industrial applications. Integrating nanosheet materials into existing membrane manufacturing processes and equipment presents challenges and opportunities, including potential material degradation, leaching, or alterations in surface properties over time, which can impact membrane lifespan and maintenance requirements [5, 19]. For the successful adoption of 2D engineered membranes in the industrial context, a thorough evaluation of operational and financial benefits is essential. Balancing the additional costs associated with nanosheet procurement and integration against the potential benefits in terms of extended membrane lifespan and improved water quality is crucial. Any modification to membrane manufacturing processes must align with environmental standards to ensure the responsible implementation of nanosheet materials, minimizing environmental risks and regulatory concerns. While our study underscores the potential benefits of MoS_2_ nanosheets in membrane manufacturing systems, addressing these opportunities is vital for their safe and effective integration into the water treatment industry.

## Conclusions

In this study, a TFN membrane was synthesized using OH–MoS_2_ nanosheets as the nanofiller (0.010 wt.%) in the aqueous monomer phase to engineer fouling-resistant membranes to handle the NOM issue. The engineered TFN membrane exhibited enhanced hydrophilicity, negative charge, and rougher membrane surface, which increased the membrane water permeance by 46.33% from 11.2 to 16.39 L m^−2^ h^−1^ bar^−1^ when compared to the control PPA membrane, while a very high salt rejection was retained at 96.3% for Na_2_SO_4_. During 6 h of filtration trials with both hydrophobic (HA) and hydrophilic (SA) fractions of NOM, the modified membrane demonstrated improved fouling resistance and NOM removal. Normalized fluxes of 95.09% and 93.26% were retained with feed water containing HA and SA, respectively. Compared to HA, SA caused more intensive fouling and flux decline in the control PPA membrane, reaching 89.71% and 74.25%, respectively. This performance was linked to the deposition of SA through hydrophilic interactions with the membrane surface. Fouling phenomena are quite complex depending upon NOM properties, its concentration and feed chemistry, and physicochemical properties of the membrane, and filtration conditions. However, engineering TFN membranes with OH–MoS_2_ endowed the membrane with favourable surface hydrophilicity, charge, and morphology, facilitating an enhanced fouling mitigation performance for both HA and SA. This study investigated the impact of Ca^2+^ and Mg^2+^ on the organic fouling and separation performance of membranes. Ca^2+^ had a more detrimental impact on organic fouling and NOM removal than Mg^2+^ for both HA and SA fractions because of its stronger affinity to neutralize and aggregate NOM molecules, as well as form complex bridging with deprotonated groups of NOM and the polymer structure of membrane, causing formation of dense foulant layer on the membrane surface. The TFN membrane outperformed the commercial and control PPA membranes during fouling tests in the presence of 1 mM Ca^2+^ and Mg^2+^ in the feed solution. Owing to the versatile performance of TFN membranes while handling various fractions of NOM and detrimental levels of alkaline earth cations, OH–MoS_2_-incorporated membranes could be favourable for the treatment of surface water containing NOM to produce potable water.

### Supplementary Information

**Additional file 1**. **Figure S1(a-d)** SEM images of OH-MoS_2_ nanosheet at various magnification. **Figure S2** FTIR spectra of bulk MoS_2_ powder, polycarbonate support membrane and OH-MoS_2_ nanosheet dispersion coated over polycarbonate membrane. **Figure S3** Cross-sectional SEM images of (a) control PPA and (b) OH-MoS_2_ PPA membrane. **Figure S4** 2D and 3D AFM images of commercial XN 45 membrane. **Figure S5** Trends of normalized flux during 6 h fouling tests conducted under different divalent cation contents and NOM feed solutions for the commercial XN 45 membrane (a–d). **Figure S6** SEM images of fouled XN 45 membrane with (a) HA + 1 mM Ca^2+^ and (b) SA + 1 mM Ca^2+^. **Table S1** Comparison of the performance characteristics of prepared membranes with other 2D enabled TFN membranes. **Table S2** Comparative performance of normalized flux and NOM removal of XN 45 and OH-MoS2 PPA membrane in the presence of detrimental levels of Ca^2+^ and Mg^2+^. **Table S3** Zeta potential of humic acid and sodium alginate solution with different concentration of Ca^2+^ and Mg^2+^ measured at pH 7. **Table S4** Relative flux recovery ratio of the membranes evaluated after water cleaning at the end of 6 h fouling experiments. **Table S5** Summary of studies evaluating impact of Ca^2+^ and Mg^2+^ on organic fouling performance of nanofiltration membranes.

## Data Availability

Available upon request.
