# Supplementary material for: Tuning nanofiltration membrane performance: OH–MoS2 nanosheet engineering and divalent cation influence on fouling and organic removal
Source: Discov Nano. 2023 Oct 23;18(1):131. doi: 10.1186/s11671-023-03909-2 (PMC10593713; doi:10.1186/s11671-023-03909-2)
Supplement: Supplementary file 1 — Additional file 1. Figure S1(a-d) SEM images of OH-MoS2 nanosheet at various magnification. Figure S2 FTIR spectra of bulk MoS2 powder, polycarbonate support membrane and OH-MoS2 nanosheet dispersion coated over polycarbonate membrane. Figure S3 Cross-sectional SEM images of (a) control PPA and (b) OH-MoS2 PPA membrane. Figure S4 2D and 3D AFM images of commercial XN 45 membrane. Figure S5 Trends of normalized flux during 6 h fouling tests conducted under different divalent cation contents and NOM feed solutions for the commercial XN 45 membrane (a–d). Figure S6 SEM images of fouled XN 45 membrane with (a) HA + 1 mM Ca2+ and (b) SA + 1 mM Ca2+. Table S1 Comparison of the performance characteristics of prepared membranes with other 2D enabled TFN membranes. Table S2 Comparative performance of normalized flux and NOM removal of XN 45 and OH-MoS2 PPA membrane in the presence of detrimental levels of Ca2+ and Mg2+. Table S3 Zeta potential of humic acid and sodium alginate solution with different concentration of Ca2+ and Mg2+ measured at pH 7. Table S4 Relative flux recovery ratio of the membranes evaluated after water cleaning at the end of 6 h fouling experiments. Table S5 Summary of studies evaluating impact of Ca2+ and Mg2+ on organic fouling performance of nanofiltration membranes. [file 11671_2023_3909_MOESM1_ESM.docx]

**Supplementary Information for manuscript**

**Title**

**Tuning Nanofiltration Membrane Performance: OH-MoS_2_ Nanosheet Engineering and Divalent Cation Influence on Fouling and Organic Removal**

**Authors**

Deepak Surendhra Mallya ^1*^, Guoliang Yang ^2^, Weiwei Lei ^2^, Shobha Muthukumaran ^3,4^ and Kanagaratnam Baskaran ^1^

^1^ School of Engineering, Deakin University, Waurn Ponds, Geelong, Victoria 3216, Australia

^2^ Institute of Frontier Materials, Deakin University, Waurn Ponds, Geelong, Victoria 3220, Australia

^3^ Institute for Sustainability Industries and Liveable Cities, Victoria University, Melbourne, VIC 3011, Australia

^4^ College of Sport, Health & Engineering, Victoria University, Melbourne, VIC 3011, Australia

**Corresponding author**

* dmallya@deakin.edu.au (Deepak Surendhra Mallya)


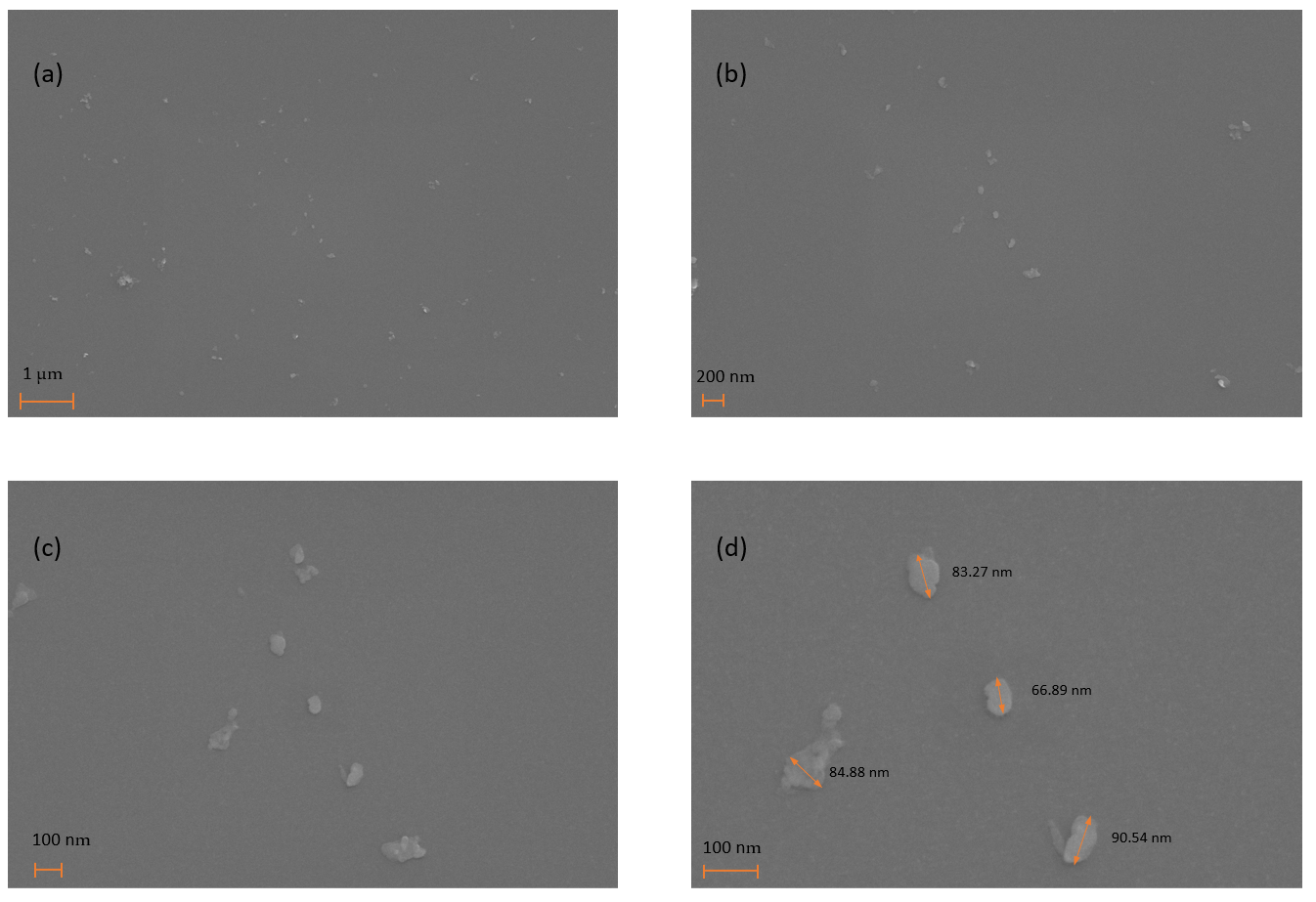


Figure S1(a-d) SEM images of OH-MoS_2_ nanosheet at various magnifications.





Figure S2 FTIR spectra of bulk MoS_2_ powder, polycarbonate support membrane and OH-MoS_2_ nanosheet dispersion coated over polycarbonate membrane.


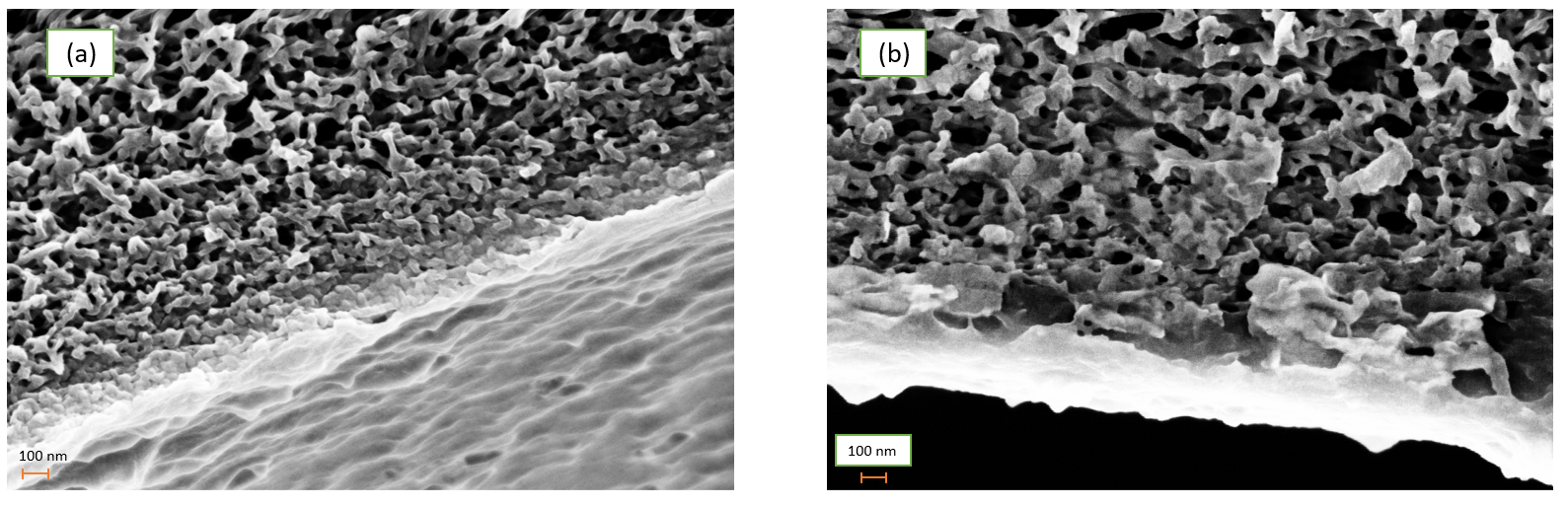


Figure S3 Cross-sectional SEM images of (a) control PPA and OH-MoS_2_ PPA membrane.


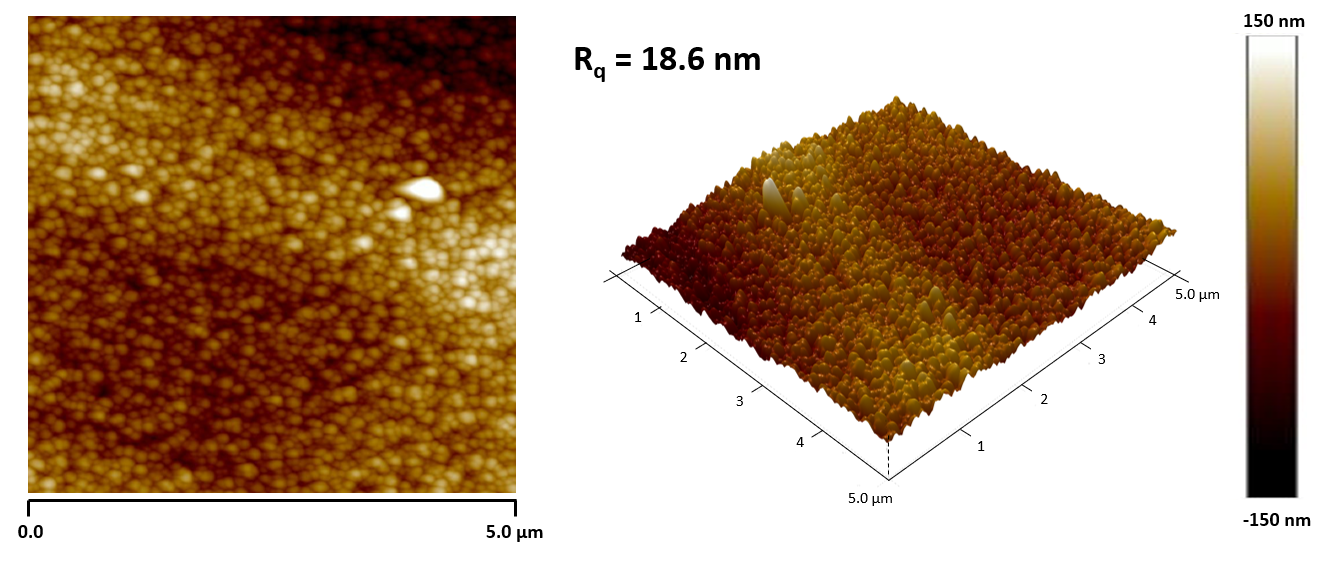


Figure S4 2D and 3D AFM images of commercial XN 45 membrane.


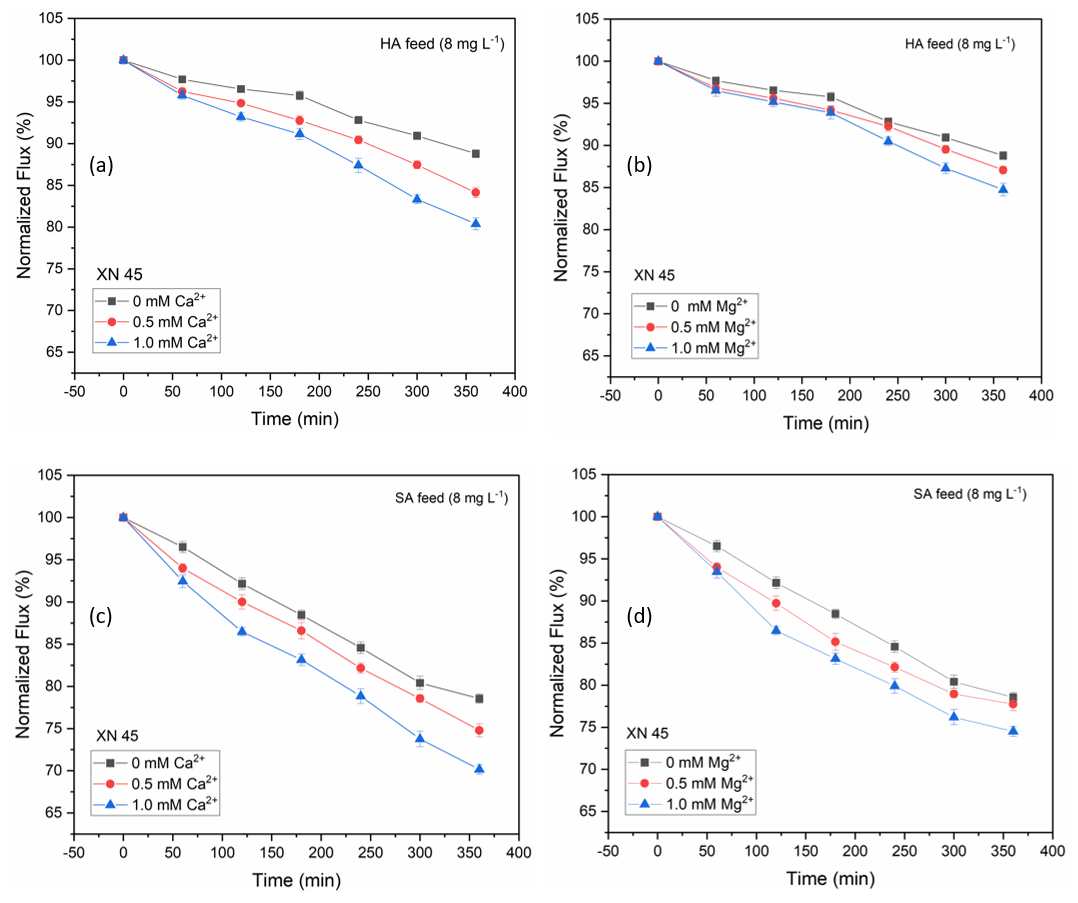


Figure S5 Trends of normalized flux during 6 h fouling tests conducted under different divalent cation contents and NOM feed solutions for the commercial XN 45 membrane **(a – d).**


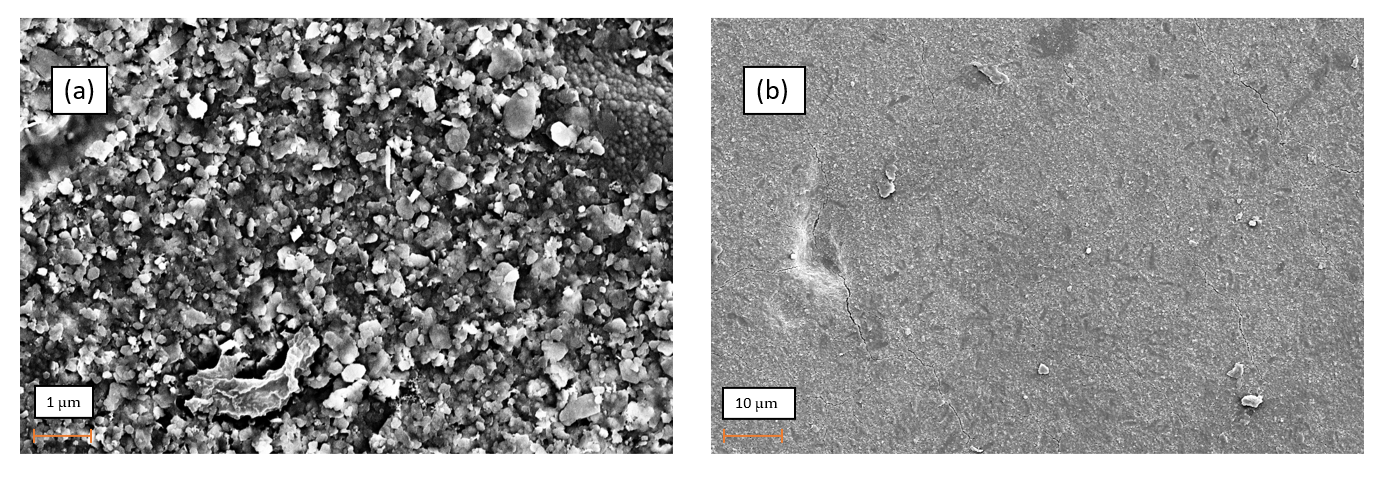


Figure S6 SEM images of fouled XN 45 membrane with (a) HA + 1 mM Ca^2+^ and (b) SA + 1 mM Ca^2+^

Table S1 Comparison of the performance characteristics of prepared membranes with other 2D enabled TFN membranes.

| S.NO | Nanosheet material | Average size (s) and thickness (T) | Optimum concentration in (x) phase | Pure water permeance of TFN membrane (L m^-2^ h^-1^ bar^-1^) | Performance enhancement (%) | Na_2_SO_4_ rejection (%) | Ref |
| --- | --- | --- | --- | --- | --- | --- | --- |
| 1 | OH-MoS_2_ | S = 60 – 90 nm  T = ~3.5 nm | 0.010 wt% (A) | 16.39 | 46.33 | 96.3 | This work |
| 2 | O-MoS_2_ | S = 100 – 500 nm  T = 3.7 nm | 0.010 wt% (O) | 7.4 | 147.62 | 97.6 | (1) |
| 3 | O-MoS_2_ | S = 100 – 500 nm  T = 1.8 – 5 nm | 0.010 wt% (O) | 7.91 | 154.34 | 97.9 | (2) |
| 3 | S-MoS_2_ | T = 4 – 5 nm | 0.019 wt% (A) | 6.78 | 35.5 | 98.8 | (3) |
| 4 | BN(NH_2_) | S = 100-200 nm  T = 1.5 – 5nm | 0.004 wt% (A) | 13.88 | 7.43 | 68.93 | (4) |
| 6 | MAH-GO | S = 400-800 nm  T = 1.3 nm | 0.006 wt% (A) | 8.22 | 76.7 | 97.6 | (5) |
| 7 | g-C_3_N_4_ | S = 300 – 400 nm  T = 1.4 – 1.6 nm | 0.010 wt% (A) | 10.2 | 37.8 | 92 | (6) |

Table S2 Comparative performance of normalized flux and NOM removal of XN 45 and OH-MoS_2_ PPA membrane in the presence of detrimental levels of Ca^2+^ and Mg^2+^.

| Membrane ID | NOM feed and concentration | Normalized flux at the end of 6 h filtration tests | DOC removal (%) |
| --- | --- | --- | --- |
| XN 45 | HA | 88.79 | 83.64 |
|  | HA + 1 mM Ca^2+^ | 80.38 | 80.67 |
|  | HA + 1 mM Mg^2+^ | 84.74 | 81.52 |
|  | SA | 78.41 | 91.06 |
|  | SA + 1 mM Ca^2+^ | 69.74 | 87.48 |
|  | SA + 1 mM Mg^2+^ | 74.58 | 89.79 |
| OH-MoS_2_ PPA | HA | 95.09 | 89.26 |
|  | HA + 1 mM Ca^2+^ | 90.52 | 86.46 |
|  | HA + 1 mM Mg^2+^ | 92.79 | 88.16 |
|  | SA | 93.26 | 93.46 |
|  | SA + 1 mM Ca^2+^ | 86.58 | 89.54 |
|  | SA + 1 mM Mg^2+^ | 92.58 | 90.71 |

Table S3 Zeta potential of humic acid and sodium alginate solution with different concentration of Ca^2+^ and Mg^2+^ measured at pH 7

| Concentration of cation (mM) | HA Feed (8 mg L^-1^) | SA Feed (8 mg L^-1^) |
| --- | --- | --- |
| 0 mM Ca^2+^ | -31.48 ± 0.5 mV | -40.5 ± 0.6 mV |
| 0.5 mM Ca^2+^ | -29.45 ± 0.4 mV | -38.86 ± 0.8 mV |
| 1.0 mM Ca^2+^ | -26.49 ± 0.9 mV | -32.49 ± 1.4 mV |
| 0.5 mM Mg^2+^ | -30.46 ± 1.1 mV | -39.4 ± 0.9 mV |
| 1.0 mM Mg^2+^ | -28.46 ± 0.7 mV | -37.5 ± 0.3 mV |

Table S4 Relative flux recovery ratio of the membranes evaluated after water cleaning at the end of 6 h fouling experiments.

| Membrane ID | NOM and cation concentration | Relative flux recovery ratio  R_re_ (%) |
| --- | --- | --- |
| Control PPA | HA | 92.25 |
| OH-MoS_2_ PPA | HA | 98.49 |
| Control PPA | HA + 1 mM Ca^2+^ | 81.54 |
| OH-MoS_2_ PPA | HA + 1 mM Ca^2+^ | 96.37 |
| Control PPA | HA + 1 mM Mg^2+^ | 88.46 |
| OH-MoS_2_ PPA | HA + 1 mM Mg^2+^ | 95.14 |
| Control PPA | SA | 95.46 |
| OH-MoS_2_ PPA | SA | 98.19 |
| Control PPA | SA + 1 mM Ca^2+^ | 80.46 |
| OH-MoS_2_ PPA | SA + 1 mM Ca^2+^ | 95.35 |
| Control PPA | SA + 1 mM Mg^2+^ | 85.46 |
| OH-MoS_2_ PPA | SA + 1 mM Mg^2+^ | 96.75 |

Table S5 Summary of studies evaluating impact of Ca^2+^ and Mg^2+^ on organic fouling performance of nanofiltration membranes

| S.N0 | Feed water characteristics and membrane setup | Inference from the study | Ref. |
| --- | --- | --- | --- |
| 1 | NOM – Humic acid (8 mg L^-1^)  – Sodium Alginate (8 mg L^-1^)  Cation – Ca^2+^ (0, 0.5 and 1.0 mM)  – Mg^2+^ (0, 0.5 and 1.0 mM)  Membrane type: OH-MoS_2_ TFN membrane  Filtration pressure: 6 bar  System: Crossflow filtration (Sterlitech – CF042) | - Presence of Ca^2+^ aggravated organic fouling through organic- Ca^2+^ complexation, reduction in membrane-foulant electrostatic repulsion and increasing foulant affinity for membrane surface. - Compared to Ca^2+^, Mg^2+^ caused less severe fouling. - Fouling by sodium alginate was more severe than humic acid. - The irreversible fouling aggravated in the presence of cations. - OH-MoS_2_ nanosheet modified membrane exhibited enhanced organic fouling resistance due to its engineered membrane surface properties. | This study |
| 2 | NOM – Sodium Alginate (200 mg L^-1^)  Cation – Ca^2+^ (0.5 mM)  – Mg^2+^ (0.5 mM)  Membrane type: NF90  Filtration pressure: 8 bar  System: Crossflow filtration (Custom made) | - Presence of Ca^2+^ aggravated organic fouling leading to dense gel layer formation reducing the normalized flux. - Compared to Ca^2+^, Mg2+ caused less severe fouling | (6) |
| 3 | NOM – Humic acid (10 mg L^-1^)  – Fulvic acid (10 mg L^-1^)  Cation – Ca^2+^ (2 mM)  Membrane type: TiO_2_ engineered NF membrane  Filtration pressure: 5 bar  System: Crossflow filtration (Custom made) | - Membrane fouling is influenced by various factors such as feed water chemistry, membrane surface properties and the NF process parameters. - HA and FA fouling in the presence of Ca^2+^ show increasing or decreasing trend depending upon the surface chemistry of membranes. - Contradictory results reported compared to the literature: hydrophilic membrane exhibited greater flux decline while hydrophobic membrane showed lower fouling. - Complexation and bridging of NOM and membrane surface in the presence of cation promoted the deposition of foulants. | (7) |
| 4 | NOM – Sodium Alginate (50, 100, 150, 200 mg L^-1^)  Cation – Mg^2+^ (0, 1, 5, 10, 50 mM)  Membrane type: Commercial membrane  Filtration pressure: 2 bar  System: Crossflow filtration | - Presence of Mg^2+^ aggravated the fouling problems during filtration. - Thick and dense gel layer formation in the presence of Mg^2+^. - Mg^2+^ enhanced the crosslinking of alginate through its interaction with carboxylic and hydroxy groups and facilitated formation of large exopolymer structures causing intense membrane fouling. | (8) |
| 5 | NOM – Sodium Alginate (1000 mg L^-1^)  Cation – Ca^2+^ (0, 0.5, 1, 1.5, 2, 2.5, 3, 4 and 6 mM)  Membrane type: Commercial PVDF membrane  Filtration pressure: 0.4 bar  System: Stirred cell dead end filtration | - Presence of Ca^2+^ in feed water significantly increased the fouling - The filtration resistance of the gel layer formed exhibited unimodal pattern with increasing content of Ca^2+^. - Initial calcium binding of alginate molecules was based on intermolecular interactions rather than intramolecular which is the key mechanism behind the changes in the micromorphology and specific filtration resistance of alginate gel with increase in Ca^2+^ content. | (9) |
| 6 | NOM – Humic acid (20 mg L^-1^)  Cation – Ca^2+^ (0, 2, 5 and 10 mM)  – Mg^2+^ (0, 2, 5 and 10 mM)  Membrane type: Commercial DK membrane  Filtration pressure: 10 bar  System: Crossflow filtration (Custom made) | - The results suggested contradictory behaviour with Mg^2+^ leading to severe flux decline and fouling compared to Ca^2+^. - Mg^2+^ ions increased the hydrodynamic colloidal size of NOM causing dense membrane fouling compared to Ca^2+^ mainly attributed to the favourable specific charge and binding ability. | (10) |
| 7 | NOM – Sodium Alginate (1000 mg L^-1^)  Cation – Ca^2+^ (0, 1.5 and 6.0 mM)    Membrane type: TFN membrane  Filtration pressure: 0.4 bar  System: Stirred cell dead end filtration | - Remarkably high specific filtration resistance (SFR)of alginate gel complexing with 1.5 mM Ca^2+^ attributed to its impact on complexation of NOM and was explained according to thermodynamics of Flory-Huggins lattice theory. - At 6 mM Ca2+ the SFR reduced due to the effects of preferential intermolecular coordination and Flory-Huggins lattice theory. | (11) |

**References**

1. Yang S, Tian H, Hill MR, Zhang K. Effect and regulation mechanism of oxidation degrees on the O–MoS2 structure and separation performance of nanofiltration membrane. Journal of Membrane Science. 2021;635:119468.

2. Yang S, Jiang Q, Zhang K. Few-layers 2D O-MoS2 TFN nanofiltration membranes for future desalination. Journal of Membrane Science. 2020:118052.

3. Zhu J, Meng W, Xue Q, Zhang K. Two dimensional sulfonated molybdenum disulfide (S–MoS2) thin-film nanocomposite nanofiltration membrane for selective desalination. Journal of Membrane Science. 2023;676:121574.

4. Abdikheibari S, Lei W, Dumée LF, Milne N, Baskaran K. Thin film nanocomposite nanofiltration membranes from amine functionalized-boron nitride/polypiperazine amide with enhanced flux and fouling resistance. Journal of Materials Chemistry A. 2018;6(25):12066-81.

5. Xie Q, Shao W, Zhang S, Hong Z, Wang Q, Zeng B. Enhancing the performance of thin-film nanocomposite nanofiltration membranes using MAH-modified GO nanosheets. RSC advances. 2017;7(86):54898-910.

6. Mahlangu OT, Mamba BB, Verliefde AR. Effect of multivalent cations on membrane‐foulant and foulant‐foulant interactions controlling fouling of nanofiltration membranes. Polymers for Advanced Technologies. 2020;31(11):2588-600.

7. Mustafa G, Wyns K, Buekenhoudt A, Meynen V. New insights into the fouling mechanism of dissolved organic matter applying nanofiltration membranes with a variety of surface chemistries. Water research. 2016;93:195-204.

8. Wang R, Liang D, Liu X, Fan W, Meng S, Cai W. Effect of magnesium ion on polysaccharide fouling. Chemical Engineering Journal. 2020;379:122351.

9. Zhang M, Lin H, Shen L, Liao B-Q, Wu X, Li R. Effect of calcium ions on fouling properties of alginate solution and its mechanisms. Journal of Membrane Science. 2017;525:320-9.

10. Song Y, Li X, Li C, Li J, Dong Z, Zhang M, et al. Exploring and comparing the roles of Ca2+ and Mg2+ in small-sized natural organics-induced charged nanofiltration membrane fouling. Separation and Purification Technology. 2020;251:117415.

11. You X, Teng J, Chen Y, Long Y, Yu G, Shen L, et al. New insights into membrane fouling by alginate: Impacts of ionic strength in presence of calcium ions. Chemosphere. 2020;246:125801.
